# Exploring EBNA3C Genetic Variability and Recombination in Epstein–Barr Virus-Associated Cancers

**DOI:** 10.3390/ijms27073054

**Published:** 2026-03-27

**Authors:** Abdiel Barra, Paulina Vasquez-Aguilar, Paulo Henrique Braz-Silva, Louise Zanella

**Affiliations:** 1 Doctorado en Ciencias Médicas, Universidad de La Frontera, Temuco 4811322, Chile; abdiel.barra@gmail.com (A.B.); p.vasquez12@ufromail.cl (P.V.-A.); 2 Medical Science Research Laboratory, Universidad de La Frontera, Temuco 4811322, Chile; 3 Diagnósticos y Evaluación Facultad de Ciencias de la Salud, Universidad Católica de Temuco, Temuco 4780000, Chile; 4 Department of Stomatology, School of Dentistry, University of São Paulo, São Paulo 05508-000, Brazil; pbraz@usp.br; 5 Institute of Tropical Medicine of São Paulo, School of Medicine, University of São Paulo, São Paulo 05403-000, Brazil

**Keywords:** herpesvirus 4, human, γ-Herpesviridae, Epstein–Barr virus nuclear antigens, lymphocryptovirus nuclear antigen 3C, recombination, phylogeny, RBPJ protein, human, Nm23 Nucleoside Diphosphate kinases, basic-leucine zipper transcription factors

## Abstract

Epstein–Barr virus is a globally disseminated oncovirus capable of causing various malignancies, including gastric cancer, Burkitt lymphoma, and Hodgkin’s lymphoma. The influence of recombination on the EBV genome revealed limitations in the current traditional EBV classification, and the extent of these recombination events across the EBV genome is not fully understood. The nuclear antigen 3C (EBNA3C) is an indispensable gene in the oncogenesis of the virus. Despite its critical role, little is known about EBNA3C sequence variability. We examined 988 EBNA3C gene sequences extracted from EBV genomes in this context. Among the protein motifs, the interaction sites with Nm23-H1, RBP-Jk, and nuclear localization signal (NLS) 2 and 3 were the most divergent between EBV types, while NLS-1 and the leucine zipper-like showed high conservation. In our study of the impact of recombination vs. point mutations in the EBNA3C gene, we found that recombination contributed five times more to substitutions than mutation. Notably, Asian populations exhibited the highest variability and recombination rates. Importantly, our analysis revealed geographical rather than disease-specific markers. Furthermore, filtering for recombination regions did not affect the classical classification of EBV-1 and EBV-2. This finding suggests that recombination is pivotal in the architecture of EBV genetic diversity of the EBNA3C gene.

## 1. Introduction

Epstein–Barr virus (EBV) is a human herpes virus that infects more than 90% of people worldwide. It is linked with a range of clinical outcomes, from mild conditions like infectious mononucleosis (IM) to several neoplastic conditions, including Burkitt lymphoma (BL), Hodgkin’s lymphoma (HL), nasopharyngeal cancer (NPC), gastric cancer (GC), and lung cancer (LC) [1,2,3]. Although EBV has a clear association with cancer, it is still neglected in public health.

Traditionally, Epstein–Barr virus (EBV) is classified into two types: type 1 (EBV-1) and type 2 (EBV-2). Recent studies have revised this classification in light of recombination, underscoring the impact of genetic reshuffling in the genomic context, as previously shown with complete genomes and the EBNA3A gene [1,4]. EBV phylopopulations have only been classified using complete genome data. The EBV genome is large and complex (due to its internal repeats and recombination regions) compared to other viruses, making sequencing challenging. However, the potential of a single gene or a combination of a limited set of genes to provide sufficient phylogenetic signal for this classification remains unexplored.

The EBV nuclear antigen 3 (EBNA3) is a family of viral proteins contributing to EBV latency and B-cell transformation. EBNA3 comprises three proteins, EBNA3A, EBNA3B, and EBNA3C, varying the significance level in the processes [5,6]. Because of the amino acid sequence homology, it is believed that these genes originated through a series of duplication events during their evolutionary history [7]. Research on recombination within the EBNA3 family has primarily focused on EBNA3A, with a lack of information available about the other EBNA3 proteins. Knockout and recombinant studies have shown that EBNA3A and EBNA3C are indispensable for establishing indefinitely growing lymphoblastoid cell lines (LCLs) in vitro [5,6]. Conversely, studies using recombinant viruses have shown that mutants lacking the EBNA3B region remain competent to transform B cells in vitro [8]. One critical interaction involves the EBNA3C and the metastasis regulatory gene Nm23-H1 [9] under certain conditions, cell proliferation has been observed even in the absence of EBNA3A [6]. This evidence highlights EBNA3C as a key to EBV latency and B-cell transformation.

EBNA3C is an essential transcriptional regulator that plays a crucial role in the EBV-mediated transformation leading to B-cell immortalization. Composed of two exons called BERF3 and BERF4, separated by an intron [10,11]. EBNA3C is a nuclear protein, and its translocation into the nucleus is facilitated by multiple nuclear localization signals (NLS) present in its sequence [12,13], which enables interaction with numerous cellular factors, like complement receptor 2 (CD21) and multiple transcription factors, such as RBP-Jκ, Cyclin A, and c-Myc, which play a pivotal role in modulating the host gene expression during the latency phase [9,14,15,16,17,18,19,20,21,22,23]. The basic leucine zipper-like (bZIP) domain further regulates transcription via DNA binding and dimerization, while NM23-H1 influences replication through its nucleotide kinase activity [14,24,25].

In this study, we analyzed EBNA3C sequences retrieved from EBV genomes to explore the influence of recombination in functional sequence variability, focusing on domain-specific variations to discuss their possible implications for B-cell transformation and tumor progression. Our results highlight the role of recombination in the evolution of EBNA3C, providing new insights into genetic diversity and its impact on virus biology.

## 2. Results

### 2.1. EBNA3C Sequences Metadata

A total of 988 EBNA3C sequences were recovered from 1314 genomes, covering a broad geographic range across Africa, America, Asia, Europe, and Oceania (see Supplementary Table S1). The dataset includes 147 sequences from Africa (East Africa, Ghana, Kenya, and North Africa), 79 from the Americas (Argentina, Brazil, and the USA), 925 from Asia (China, Indonesia, Japan, Korea, Singapore, South Korea, Taiwan, and Vietnam), 133 from Europe (Finland, France, Germany, Poland, Ukraine, and the United Kingdom) and 30 from Oceania (Australia and Papua New Guinea). Isolates from Asia, particularly China and Japan, are overrepresented, constituting 70% of the total. In contrast, sequences from Oceania and South America are underrepresented. The health statuses of EBV-infected patients varied, with 19% classified as healthy individuals and 81% diagnosed with verified EBV-associated diseases. Associated diseases were angioimmunoblastic T-cell lymphoma (AIL), AIDS-related lymphoma (ARL), BL, chronic active EBV infection (CAEBV), diffuse large B-cell lymphoma (DLBCL), EBV-related disease (EBV-RD—no precise disease condition described), GC, HL, IM, LC, lymphoblastoid cell line (LCL), lymphoepithelioma (LE), lymphoid neoplasia (LM), natural killer T-cell lymphoma (NKTCL), NPC, post-transplant B-cell lymphoma (PTBL), post-transplant lymphoproliferative disorder (PTLD), lymphoepithelioma-like carcinoma of the lung (LELC), spontaneous lymphoblastoid cell line (sLCL), and T cells disorders (TCDs).

### 2.2. Recombination Rates and Recombination-Induced Motifs Distribution in the EBNA3C Gene

The impact of recombination events and point mutations was estimated using ClonalFrameML v.1.13. The average length of recombination fragments was δ = 16 bp [14.6–16.9], and the average divergence between the donor and the recipient ν = 0.222 [0.218–0.237]. The ratio of rates of recombination and mutation was p/θ = 1.395 [1.290–1.500], whereas the ratio of effects of recombination and mutation was r/m = 5 [4.64–5.38]. This indicated that recombination occurs ~1.4 times more often than mutation, and because each recombination event introduced on average δν = 3.6 substitutions, recombination overall caused five times more substitutions than mutation, confirming the substantial role of recombination in EBV evolution.

The presence and distribution of specific recombination initiation motifs were analyzed. We found the CCCAG motif significantly enriched within EBV1 recombinant regions, highlighting its role as a potential recombination initiator. Additional motifs, including AGGAG and GGGCT, appear within these recombination regions. Genotype differences in motif abundance were observed: EBV2 showed more CCCAG (14 vs. 12 in EBV1), but fewer GGGCT (2 vs. 6) and TGGAG (3 vs. 4) motifs than EBV1.

### 2.3. Phylogenetic Reconstruction of EBNA3C

**Figure 1:**
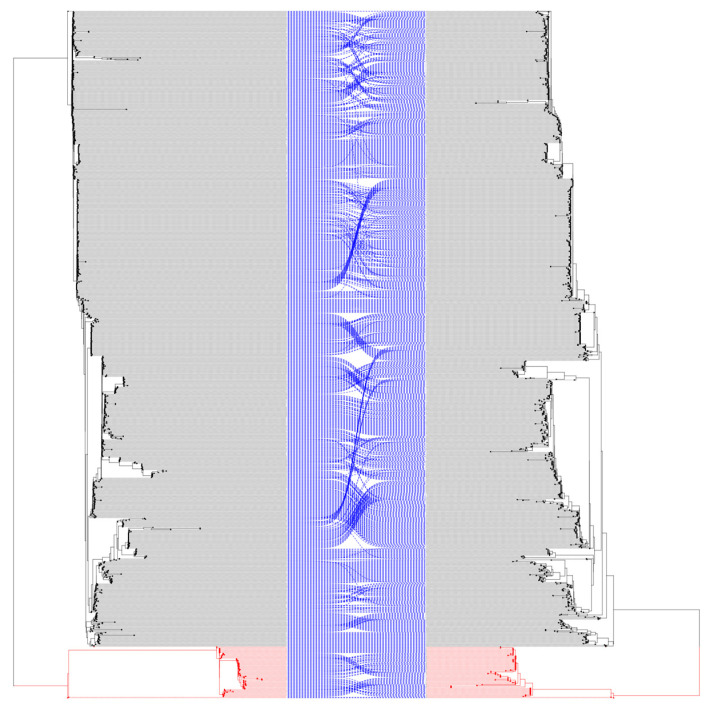
Phylogenetic comparison of EBNA3C unfiltered (**left**) vs. filtered (**right**) recombination regions. Phylogenetic reconstruction of EBNA3C includes both exons (BERF3 and BERF4) and excludes the intron. The left tree shows the reconstruction with unfiltered recombinant regions, and the right tree shows the reconstruction with recombinant regions removed. The red branches of the tree indicate the EBV-2 isolates. The black dots represent the EBV isolates. The lines connect the identical isolates in the different trees. Some isolates exhibit internal rearrangements within their phylogenetic clade, which are demonstrated by dashed blue lines.

A previous study highlighted the impact of recombination in EBV by comparing phylogenetic reconstructions of complete genomes with and without filtering recombination [1]. Considering these findings, we applied a similar approach to the EBNA3C gene. Phylogenetic reconstructions of EBNA3C, excluding the intronic region, were inferred from both unfiltered and recombination-filtered alignments to assess the impact of recombination on phylogenetic inference. The tree topologies were compared to assess the impact of recombination in the EBNA3C gene. Both unfiltered and recombination-filtered trees showed identical topology with maximum bootstrap support for the split EBV-1 (914 sequences) and EBV-2 (74 sequences) (see Figure 1, Supplementary Figure S1a,b). No significant variations in the relationship among isolates were observed within EBV-1 and EBV-2 genotypes after recombinant filtering. Only minor branch rearrangement was observed. Both EBV-1 and EBV-2 genotypes exhibit internal rearrangements; however, no intermixing between genotypes was identified.

### 2.4. EBNA3C Phylopopulations Estimation

**Figure 2:**
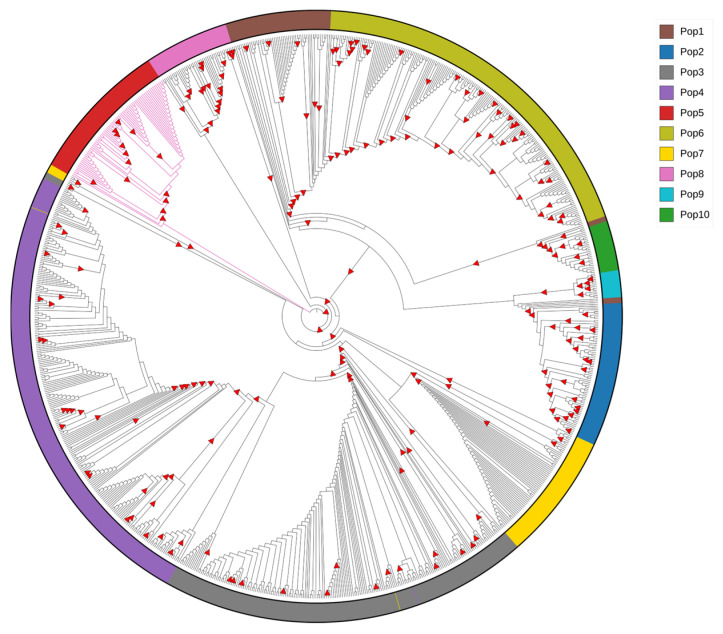
Phylogenetic reconstruction of EBNA3C with filtered putative recombinant regions. The maximum likelihood tree includes BERF3 and BERF4 exons. Isolates with pink branches correspond to EBV-2. The outer circle shows the 10 distinct populations defined through hierarchical Bayesian clustering. The legend represents the corresponding color for each 10 populations. Bootstrap values above 80% are shown with red triangles.

Phylogenetic analysis using RhierBAPS revealed 10 distinct EBV populations at the first hierarchical level. Each population showed distinct patterns of geographic distribution and disease manifestation, including non-malignant manifestations and diverse cancer types. However, this geographic pattern may reflect sampling bias, as data were only available from China, Kenya, the United States, and the United Kingdom (see Supplementary Table S1). Similarly, the relationship of EBV and different health conditions varied among populations, emphasizing the complexity of the EBV population structure. Regarding the phylogenetic relationships of the ten populations analyzed, five (Pop2, Pop5, Pop7, Pop8, and Pop10) exhibit a monophyletic structure, and the other five populations (Pop1, Pop3, Pop4, Pop6, and Pop9) are paraphyletic (see Figure 2).

### 2.5. Identification of Putative Recombinant Regions in EBNA3C

**Figure 3:**
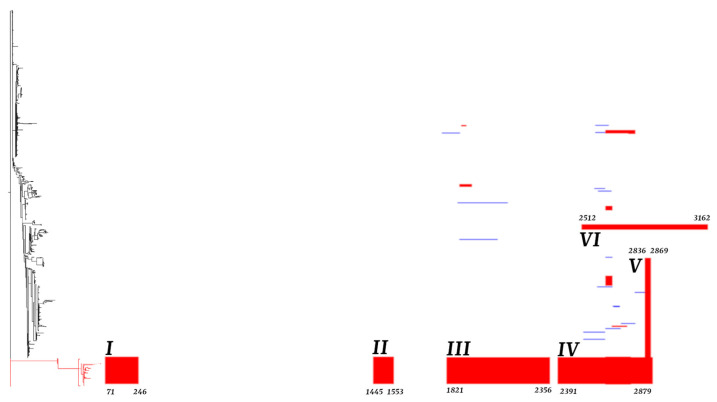
Predicted putative recombinant regions in EBNA3C. The figure depicts the putative recombinant regions within the EBNA3C gene. The left side of the figure shows the maximum likelihood phylogenetic reconstruction of the EBNA3C gene, excluding the intronic region. The red branches of the tree represent the sequences classified as EBV-2. On the right-hand side of the figure, red horizontal bars represent the putative recombinant regions on internal branches shared among multiple isolates through common ancestry. Each putative recombinant region was numbered from I to VI. Soft light blue horizontal bars represent putative recombinant regions specific to individual isolates. The numbers displayed at the lower edges of the horizontal bars represent the nucleotide positions of the reference sequence.

The distribution of putative recombinant regions in EBNA3C was analyzed to assess their contribution to genetic diversity. A detailed analysis of putative recombinant regions identified by Gubbins for all EBNA3C isolates revealed a limited number of putative recombinant regions within the EBV-1 clade, while a significant putative recombinant region was observed among EBV-2 isolates (see Figure 3, red horizontal bars).

Six putative recombinant regions were identified: region I (71–246), region II (1445–1553), region III (1821–2356), region IV (2391–2879), region V (2836–2869), and region VI (2512–3162). Interestingly, all isolates forming regions I, II, III, and IV belong to EBV-2 genotypes, including two isolates (HS039 and NPCT025) from the EBV-1 type. These putative recombinant regions in EBV-2 were mainly represented by China (47%) and Kenya (26%); other minor proportions were represented by France (6%), Indonesia (5%), Taiwan (4%), Ghana (3%), the United States (3%), Japan (3%), South Korea (2%) and the United Kingdom (1%). Regions V and VI belong exclusively to the EBV-1 genotype. Region V represents the clade predominantly composed of isolates from China (38%), Japan (22%), and the United States (12%) and a small representation from the United Kingdom (7%), Kenya (4%), France (4%), Argentina and Indonesia (2% each), and other countries contributing 1%: Brazil, Germany, Ghana, Italy, Poland, Singapore, South Korea, Taiwan, and Vietnam. Region VI represents a clade containing isolates from Kenya (78.6%), the United Kingdom (14.4%), and Brazil (7%). Some unique putative recombinant regions, each exclusively found in a single isolate, were identified and belong to both genotypes EBV-1 and EBV-2, as shown in Figure 3, as light blue horizontal bars. Putative recombination events defined by RDP5 are detailed in Supplementary Tables S2 and S3. 

### 2.6. EBNA3C Sequence Variation Context

**Figure 4:**
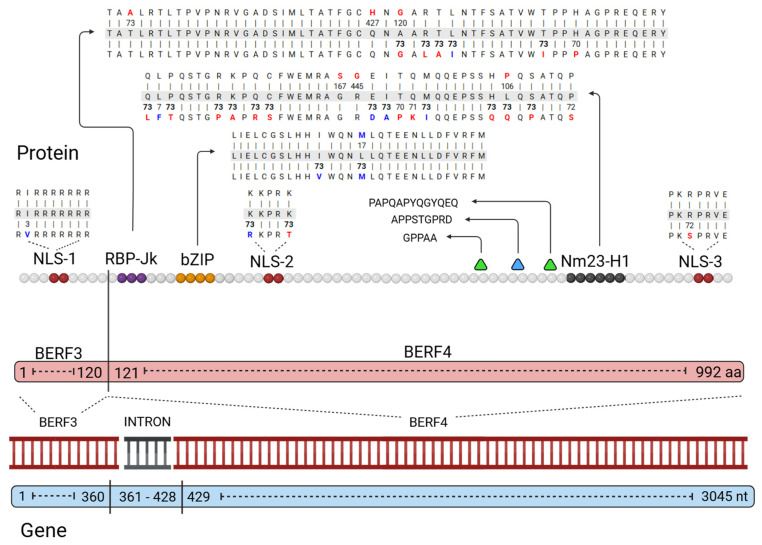
Schematic representation of EBNA3C’s sequence variation. Visualization of nucleotide and amino acid variation in EBNA3C. The two exons (BERF3 and BERF4) and the intron are depicted in the bottom part of the image, with the nucleotide (nt) size indicated inside the light blue box. The upper region of the figure indicates the functional motifs (3 NLS, RBP-JK, bZIP, and NM23-H1) concerning the correlated exon, with the amino acid (aa) size indicated inside the light red box. The amino acid substitutions identified in functional motifs indicated by arrows are depicted in three sequence lines: the reference sequence in the center, shaded with light gray; EBV-1 in the upper line; and EBV-2 in the lower line. The vertical lines between the sequences indicate identical amino acids. The numbers between the vertical lines account for the number of sequences with the indicated substitution. Conservatively substituted amino acids are shown in blue, while those with physicochemical changes are highlighted in red. The green triangles show the repetitive region characteristic of EBV-1, and the blue triangle shows the repetitive region of EBV-2.

We investigated the sequence variation in EBNA3C in the BERF3 and BERF4 exons as well as the intronic region. We found variability in all three regions, including the functional motif and NLS (see Figure 4). The analysis identified amino acid substitution patterns with specific geographic and clinical associations (see Table 1).

We identified the amino acid substitution R11I + N21D + R44G + Y51D + T107I in BERF3 as a dominant pattern associated with NPC (62.6%) in Chinese populations (99.4% of cases). The I141V + Q213H + E336D + I348L + R656G + E701Q pattern found in BERF3 of EBV-1 showed NPC association (64.8%, *p* < 0.001) in Chinese populations (99.4%, *p* < 0.001); see Table 1. Additionally, the amino acid substitution A162V + S557L + G655S + T677M + A683V + E701Q + Q740P + Q744R + P753Q + L866S + K976E, present in BERF4 of EBV-1, was the most frequent pattern among those associated with EBV-related diseases. This pattern was significantly associated with CAEBV cases (*p* < 0.001), occurring in 65.2% of samples overall and reaching 100% prevalence in Japanese CAEBV patients (see Table 1). The deletion pattern spanning regions 404–434, 680–702, 713–731, and 818–824, found exclusively in the EBV-2 genotype, was present in 84% of Burkitt lymphoma cases, all of which originated from Kenya (see Table 1).

Beyond these disease associations, our analysis revealed (i) the health-associated pattern I348L + L669P + S690P + E701Q + H831Y + C915W in BERF4 of EBV-1 in asymptomatic carriers (*p* < 0.001) from China and Indonesia, and (ii) the geographic-associated pattern R11I + N21D + R44G + Y51D in BERF3 of EBV-1 shared between patients with CAEBV (*p* < 0.001) from Japan (*p* < 0.001) and GC patients from Asia (*p* < 0.001) (see Table 1). Notably, none of the EBV-2-related sequences exhibited amino acid variation patterns in BERF3.

Beyond identifying amino acid variation patterns and deletions found in EBNA3C, we also examined repeat sequences. In BERF4, two distinct repeated clusters were observed: one consisting of GPPAA (see Figure 4, first green triangle, positions 440–489) and another composed of PAPQAPYQGYQEQ (see Figure 4, second green triangle, positions 594–686). The GPPAA motif exhibited a range of 1 to 18 repeats. The nine-repeat pattern (present in 192 sequences) was the most frequent in the Chinese population, accounting for 70.8% of NPC and 15.6% of healthy individuals. The eight-repeat pattern (present in 188 sequences) was also common in 19.7% of IM cases from the USA (97.3%), 17.6% of BL cases from Kenya (66.7%), and both NPC and healthy individuals (20.7% each) from China (77% and 100%, respectively). The PAPQAPYQGYQEQ motif ranged from 1 to 9 repeats, with 2 repeats being most common (371 sequences), mainly observed in NPC (53.1%) and healthy individuals (18.3%) in China, with lower frequencies in BL (10.8%) from Kenya, and pLELC (6.7%) from China. Additionally, the seven-repeat pattern (present in 111 sequences) was predominantly found in CAEBV (53.2% of this group) and NKTCL (14.4%), both predominantly in Japan.

A distinct feature was identified exclusively in EBV-2: a 9-amino acid repeat cluster of APPSTGPRD (see Figure 4, light blue triangle), located at positions 492 to 530 of the BERF4 amino acid sequence. This motif varied from 2 to 10 repeats, with a five-repeat configuration (36%) being the most common, particularly in BL (47.2%) from Kenya. In contrast, the four-repeat pattern was primarily observed in healthy individuals (70.6%) from China, while the three-repeat (11 sequences) was mainly found in healthy individuals (54.5%) in China as well.

Remarkably, a type-specific marker was identified, characterized by a change in the A1T intronic sequence in all EBV-2 isolates. Furthermore, the C6A change had an overall frequency of 15.2% (143 occurrences), mostly concentrated in China (50.3%) and Japan (43.4%). Among Chinese isolates, 19.4% were present in GC, while 29.2% were observed in healthy individuals. In Japan, 58.1% of C6A occurrences were linked with CAEBV.

### 2.7. Variations in EBNA3C Functional Motifs

Analysis of EBNA3C functional motifs of bZIP, NLS, RBP-Jk, and Nm23-H1 revealed the variability of these elements (Figure 4 and Table S4). The NLS (NLS-1, NLS-2, and NLS-3) showed complete conservation among EBV-1 isolates (see Figure 4, red circles). In EBV-2 isolates, the NLS-1 was identical to that of EBV-1, except for three specific EBV-2 sequences from China (HKHD134, HKNPC45, HKHD67), which exhibit the I73V conservative change. In NLS-2, all 73 EBV-2 sequences exhibited simultaneously a K414R conservative and a K418T non-conservative substitution. The NLS-3 of almost all EBV-2 sequences displayed an R941S non-conservative change, with the single exception of the HC-0004 isolate from Kenya.

The RBP-Jk interaction domain (186–240) in EBV-1 (Figure 4, purple circles) exhibits a non-conservative T188A substitution in 73 sequences (8%). This pattern was related to healthy individuals (9.6%), predominantly in the United Kingdom (85.7%), and TCD cases (9.6%) exclusively from France (100%). It was also observed in IM (20.5%), mainly from the USA (93.3%), and CAEBV (9.6%) cases, all from the United States of America (100%). A notable association was observed with BL (27.4%), primarily in Kenya (55%), with other occurrences reported in other parts of East and North Africa, Ghana, France, and Brazil. Another frequent non-conservative substitution, Q213H, was identified in 427 sequences (46.2%), predominantly in NPC cases (52.6%) and healthy individuals (19%) in China. Additionally, a non-conservative A215G change was present in 120 sequences (13.1%), related to TCD cases (6.7%) from France (100%) and IM (24.2%), predominantly in the United States of America (96.6%). This substitution was also linked to BL (29.2%), with a high prevalence in Kenya (62.9%), and present in East and North Africa, Ghana, Brazil, France, and the United States of America. We further identified co-occurrence of T188A and A215G substitutions in 57 sequences (6.2%). Among the 73 EBV-2 sequences analyzed, all harbored the non-conservative substitutions A215G, R217L, T218A, and T228I, as well as the conservative change at L219I. The L219I conservative substitution was observed in all 73 EBV-2 sequences. Regarding the H231P non-conservative change, 70 out of 73 EBV-2 sequences carry this change, while three isolates (HKHD134, HKNPC45, HKHD67) from China are identical in sequence to EBV-1. Furthermore, these three isolates exhibit characteristic patterns of conservative substitutions at N197Q, D202E, I204V, alongside N214S, and non-conservative substitutions at S203I and M205R.

In the bZIP domain (Figure 4, yellow circles), regarding the EBV-2 sequences, we can observe I273V and L277M conservative substitutions in all 73 isolates. These substitutions were predominantly found in healthy individuals (35%), with occurrences in China (80%) and Kenya (11.5%). Additionally, these variants were related to BL (27%) in Kenya (80%). The EBV-1 exhibits an L277M synonymous substitution that was identified in 17 sequences (1.9% of the total), with a notable prevalence among BL patients (80%), a high occurrence in Kenya (93%), and a small proportion in Brazil (7%).

Analysis of the Nm23-H1 motif (637–675) revealed specific substitutions, highlighting significant differences in EBV-2 compared to EBV-1 (Figure 4, graphite gray circles). The analysis shows low variations affecting the Nm23-H1 motif in EBV-1 compared with EBV-2. The overall identity of EBV genotypes was 65%, with a total of 19 substitutions. Of these, 4 were conservative (L638F, E689D, I690A, and M693I) and 15 were non-conservative (Q637L, P639T, R644P, K645A, Q647R, C648S, G655S, R656G, T659P, Q660K, H668Q, L669P, L669Q, S671P, P675S).

A total of three non-conservative substitutions were identified in EBV-1 (G655S, R656G, and L669P). The non-conservative change G655S in 167 sequences (17.7%) was significant in CAEBV (30.9%), only in Japan, and in healthy individuals (22.8%), mostly in China (92%). The R656G non-conservative change showed the highest overall frequency, 46.3% (445 sequences), being most prevalent in NPC (57.0%), mostly in China (96%) and healthy individuals (21.7%), predominantly in China (92%). Finally, the change L669P in 106 sequences (10.1%) was prominent in healthy individuals (43.5%) in China (97.5%) and CAEBV (21.7%), entirely in Japan.

In EBV-2, a total of 16 substitutions were identified, including 4 conservative (L638F, E689D, I690A, and M693I) and 12 non-conservatives (Q637L, P639T, R644P, K645A, Q647R, C648S, T659P, Q660K, H668Q, L669Q, S671P, P675S). Among the four conservative substitutions, three (E689D, I690A, and M693I) were present in all 73 EBV-2 isolates. Among the 12 non-conservative substitutions, 9 (Q637L, R644P, K645A, Q647R, C648S, T659P, H668Q, L669Q, S671P) were present in all 73 EBV-2 isolates. These 73 isolates included samples from healthy individuals (35.6% of the total) and from BL cases (27.4%), followed by ND cases with no available clinical information (19.2%), and less frequent categories such as NPC (5.5%), LCL (4.1%), TCD (2.7%), pLELC (2.7%), GC (1.4%), and NKTCL (1.4%). Geographically, isolates originated mainly from Kenya (38.4%) and China (34.2%), with smaller proportions from the USA (4.1%), the UK (2.7%), Japan (2.7%), Indonesia (2.7%), France (2.7%), and PNG (2.7%). A few additional isolates came from Taiwan, Ghana, South Korea, Singapore, and regional designations such as Africa and North Africa (1.4% each). One isolate had no geographic information available (ND). The only synonymous substitution that was not present in all EBV-2 isolates but occurred in seven (AH Saliva 9316, HKHD41, HKHD72, HKHD79, HKHD97, HKHD104, and HKHD141) of them was L638F. The L638F substitution was identified in seven sequences (9.6%), which were represented by 100% of healthy patients’ sequences from China (85%) and Taiwan (15%). We identified non-synonymous substitutions that were not present in all EBV-2 isolates but occurred in 70, 71, and 72 of them, respectively: T659P, Q660K, and P675S. The T659P substitution was observed in healthy individuals (34%) in China (75%) and BL patients (28.6%) in Kenya (80%). Similarly, the Q660K substitution is present in healthy individuals (36.6%) in China (80%) and BL patients (25.4%) in Kenya (88.9%). Finally, the P675S substitution was present in healthy individuals (34.7%) in China (80%) and BL patients (27.8%) in Kenya (80%).

### 2.8. Variability of Epitopes Among EBNA3C

The eight previously identified EBNA3C epitopes (EENLLDFVRF, EGGVGWRHW, FRKAQIQGL, KEHVIQNAF, LDFVRFMGV, LRGKWQRRYR, QPRAPIRPI, and RRIYDLIEL) were analyzed to assess their variability. EGGVGWRHW, EENLLDFVRF, and LDFVRFMGV epitopes remained completely conserved in all EBV. The results are detailed in Table 2.

In the FRKAQIQGL epitope, one conservative and three non-conservative substitutions were identified. The conservative substitution I348L and the non-conservative substitution I348M were found in EBV-1 isolates. In EBV-2 isolates, the non-conservative substitutions R344L and I348R co-occurred. The conservative substitution I348L, which had an overall frequency of 55.8%. This change was predominantly observed in NPC (46.7%) and healthy individuals (25.3%) in China. Additionally, the I348M substitution was detected with a lower overall frequency of 1.5%, showing a strong association with BL (85.7%, *p* < 0.001), primarily in Kenya (92%, *p* < 0.001). The co-occurrence of two non-conservative substitutions, R224L and I228R, was identified in all EBV-2 sequences.

In the KEHVIQNAF epitope, one conservative and three non-conservative substitutions were identified. The conservative substitution E336D and the non-conservative substitution H337Q were found in EBV-1 isolates. In EBV-2 isolates, the non-conservative substitutions H337Q and N341K co-occurred. The conservative substitution E336D was identified at a high overall frequency of 46.8%, predominantly in NPC (51.6%) and healthy individuals (20.3%) in China. Additionally, the non-conservative substitution H337Q, detected at a lower overall frequency of 1.5%, exhibited association with BL (85.7%) and was primarily found in Kenya (92.7%). The co-occurrence of two non-conservative substitutions, H337Q and N341K, was identified in all EBV-2 sequences.

In the LRGKWQRRYR epitope, a unique non-conservative substitution, Y257F, was exhibited in EBV-2 isolates. This conservative substitution was identified in healthy individuals (35%) in China (80%) and Kenya (11.5%), as well as in BL (27%), predominantly in Kenya (80%).

In the RRIYDLIEL epitope, one conservative and two non-conservative substitutions were identified. The conservative substitution R259K and the non-conservative substitution Y261F were found in EBV-1 isolates. In EBV-2 isolates, a non-conservative substitution, Y261F, was identified. The R259K conservative substitution found in EBV-1 isolates showed an overall frequency of 3.2%, occurring in small numbers across diverse conditions. The most notable occurrences were in healthy individuals (20.7%) exclusively from the United Kingdom, IM (20.7%) entirely in the United States of America, and PTLD (13.8%) all in the United Kingdom. The Y261F non-conservative change (2.4%) found in EBV-1 isolates was associated with NPC (50.0%) in Indonesia (91%, *p* < 0.001). In contrast, the Y261F non-conservative change identified in EBV-2 isolates was identified in healthy patients (35%) in China (80%) and BL patients (27%) in Kenya (80%).

Finally, the QPRAPIRPI epitope was completely conserved in all EBV-1 isolates. On the other hand, 19% of the EBV-2 isolates presented the co-occurrence of non-conservative substitutions Q761P and R763P. These non-conservative substitutions were identified in healthy individuals (14%) and in BL patients (20%) in Kenya (100%). Considering that a proline amino acid is located before the epitope, changes in these sequences lead to the presence of two additional prolines, resulting in a proline cluster.

### 2.9. Geographic and Clinical Associations of EBNA3C Mutations

In EBV-1, the strongest statistical associations were observed for NPC with R11I, N21D, R44G, Y51D, T107I, I141V, Q213H, E336D, I348L, E701Q, H782Q showing the most significant association (*p* < 0.001). For BL, the most significant associated mutations included D102G, T188A, Q213K, A215G, R217Q, L277M, I348M, T356M, T821A, A840S, L869M, A978S (*p* < 0.001). Robust associations were also found for IM (L45F, Q60P, T221S, R255K, S385T, V489A, R499I, G783R, M904L, A921S, all with *p* < 0.001), CAEBV (T104A, T107V, A162V, S557L, T677M, A683V, H782P, L866S, K976E, A978V, all with *p* < 0.001) and statistically weaker associations for GC (R44I, *p* < 000.1 only for FDR) and DLBCL (P919T, *p* < 0.001 only for FDR). Additionally, variants such as R259K, G357V, L669P, S690P, H831Y, C915W, and P916S (*p* < 0.001) were detected in the healthy control group, suggesting they represent non-pathogenic polymorphisms present in the general population. Strong geographic associations were observed in populations from the United States, with mutations T188A, A215G, T356M, V489A, A840S, M904L, and A921S (*p* < 0.001), and from Indonesia, with T104P, Y257F, and Y261F (*p* < 0.001). The Chinese population exhibited the highest number of geographically specific variants, including R11I, N21D, R44G, Y51D, T104A, T107V, I141V, A162V, Q213H, E336D, I348L, G357V, E359A, L669P, E701Q, H782Q, G783R, H831Y, L866S, C915W, P916S, and P919T (all with *p* < 0.001), with T107I being the most statistically significant (*p* < 0.001). Distinctive and statistically robust mutational profiles were identified in Kenya with D102G, R217Q, L277M, I348M, T821A, L869M, and A978S (all with *p* < 0.001), in the United Kingdom with L45F, Q60P, T221S, R255K, R259K, S385T, and R499I (all with *p* < 0.001), and in Japan with S557L, T677M, A683V, H782P, K976E, and A978V (all with *p* < 0.001). When integrated, these findings revealed a consistent pattern of mutations associated with specific pathologies. The statistically significant variants associated with NPC, including R11I, N21D, R44G, Y51D, T107I, I141V, Q213H, E336D, I348L, E701Q, and H782Q (*p* < 0.001 each), coincide with those identified as geographically specific to the Chinese population. Similarly, mutations linked to BL show strong geographical stratification. T188A, A215G, T356M, and A840S are predominantly associated with the United States, while D102G, R217Q, L277M, I348M, T821A, L869M, and A978S are characteristic of the Kenyan population.

For EBV-2, a strong association was observed for the P537L, A547T, and T553M mutation cluster in African populations (*p* < 0.001). Furthermore, another limited association was found for the T524M and I827L variants in Indonesia (*p* < 0.002), and for the G472E, F806V, and L881S variants in China (*p* < 0.009). The remaining mutations analyzed, mainly in Kenyan and Chinese populations, showed no statistically significant association. Regarding their association with clinical manifestations, EBV-2 mutations lacked statistical association to be correlated with a health status.

## 3. Discussion

Epstein–Barr virus (EBV), a global oncovirus associated with B-cell carcinomas and neoplasms, is traditionally classified into EBV-1 and EBV-2 based on their ability to transform lymphoblastoid cell lines. Recent studies suggest this classification may reflect artifacts from genomic recombination regions. Therefore, analyzing the genome with the putative recombinant regions excluded better clarifies its population structure [26]. Although this approach has only been applied to whole genomes, its implications for individual genes remain unexplored. Among viral proteins, the EBNA family regulates viral transcription, apoptosis, and host genes [26]. EBNA3C, a member of the EBNA3 family, presents a conserved genetic structure (short and long exons separated by an intron), suggesting an evolutionary origin by gene duplication [14]. While EBNA3A shows high susceptibility to recombination, EBNA3C, despite its relevance in B-cell transformation, has not been studied in this context. Unlike EBNA3B, whose absence does not affect this capacity [14]. Given EBNA3C’s key role in B cell transformation and gene regulation and its poorly understood genetic diversity, we analyzed the genetic variability and recombination patterns in 988 EBNA3C sequences to explore their association with EBV-related diseases.

Our analysis revealed a substantial bias in the geographic distribution of the sequences [27,28], with a high proportion of sequences from the Asian continent associated with NPC. Despite the recent increase in deposited EBV genomes, a notable limitation persists in the available data, particularly a lack of global representativeness. The imbalanced representation of EBV genomes from specific geographic regions introduces challenges that complicate the interpretation of disease-specific patterns [27]. This inequality in sequence distribution can be attributed to both the virus’s natural geographic spread and disparate access to sequencing resources. Moreover, the predominance of complete genomes from complex EBV-related diseases, such as NPC, suggests a prioritization of sequencing efforts toward clinically significant cases. The restricted presence of EBV-2 in certain regions may result from differences in genotype-specific viral replication efficiency, influencing its capacity for broader dissemination [29]. Phylogenetic reconstruction of EBNA3C with and without recombinant filtering confirms a higher prevalence of EBV-1 versus EBV-2, aligned with previous findings on genome-wide studies, EBNA3A, and lytic genes [1,4,30,31,32,33,34].

The EBNA3C gene showed a high recombination rate, with the calculated impact showing that recombination events contributed five times more to genetic diversity substitutions than mutations. This confirms previous genome-wide studies with a sample size, which reported a high recombination signal in the EBNA3 region, specifically in EBNA3C [1,35,36]. Previous studies using complete genomes demonstrated that the impact of recombination could be observed when comparing phylogenetic reconstruction with and without recombination regions. We compared the phylogenetic reconstruction of EBNA3C with and without recombination but were unable to identify rearrangements between EBV genotypes, detecting only internal rearrangements. Consequently, we aimed to recover previously identified EBV phylopopulations using a single-gene strategy.

Obtaining the complete EBV genome remains a costly and complex process. In this study, we aimed to assess whether EBNA3C carries sufficient phylogenetic signal to resolve EBV phylopopulations. Previous analyses identified 12 EBV phylopopulations using complete genomes, of which seven were monophyletic and five paraphyletic. The EBNA3C gene was analyzed using the HierBAPS algorithm, which allows the identification of EBV clusters. Those clusters identified herein correlate with the populations previously classified by Zanella et al. [1], who applied a similar methodology in a genomic approach. Our EBNA3C analysis identified 10 populations: five with a monophyletic structure and five with a paraphyletic structure. Our dataset included more sequences than the previous genome-based study, yielding fewer distinct populations but a higher number of paraphyletic groups. Comparison of the phylopopulations revealed similarities with whole-genome analyses, with both EBVpop1 and EBVpop2 (identified in this study) including isolates from diverse geographic regions and being associated with pathologies such as BL and IM. However, EBNA3C alone did not provide sufficient phylogenetic signal to robustly segregate EBV phylopopulations.

Furthermore, our analysis revealed that recombination regions were predominantly localized in EBV-2 isolates (Table S5), mirroring patterns previously reported for EBNA3A [4]. Unlike previous studies, we identified recombination regions within EBV-1 lineages. This is consistent with observations in other herpesviruses, where recombination facilitates adaptive divergence in geographically structured populations [37,38,39,40,41,42]. Notably, we detected two EBV-1 sequences that shared a recombination region with all EBV-2 isolates. This interchange of recombinant regions between genotypes impacts the current EBV population structure. It may also reflect an ancestral intertype recombination region that maintained minimal divergence from the EBV-1 parental lineage. This phenomenon is documented in herpesviruses under selective pressures to preserve essential gene functions [43,44,45].

Analysis of the EBNA3C intronic region revealed remarkable phylogenetic conservation between EBV-1 and EBV-2 types, with variation limited to a single change in the first nucleotide A1T. This pattern of intronic conservation is consistent with previous studies on EBNA3 family genes [4,46], where intron retention has been proposed as a post-transcriptional regulatory mechanism to modulate the expression of these viral oncoproteins [46,47,48]. The observed conservation suggests strong selective constraints are possibly linked to: (i) the presence of critical regulatory elements in introns (e.g., microRNA binding sites or splicing factors) [49], and (ii) the need to maintain overlapping open reading frames (ORFs) in compact herpesvirus genomes [47].

Several epitopes related to various processes, such as interaction with cellular proteins, regulation of the viral cycle, and other functions, have already been identified in EBNA3C [50]. However, there is still no clear characterization of genotypic variation among these epitopes, nor is it well established whether these changes have a geographic association or correlate with an increased frequency of clinical manifestations linked to EBV infection. Our analyses provide a comprehensive view of EBNA3C epitope variability. The I38L substitution in the FRKAQIQGL epitope and the E336L substitution in the KEHVIQNAF epitope could be related to NPC in China. Furthermore, the FRKAQIQGL epitope has also been linked to BL in Kenya. Specifically, this association appears when the epitope presents the I348M substitution in EBV-1 and the co-occurrence of R344L + I348R in EBV-2. The conservation of these substitutions, coupled with their possible role as disease markers in some geographic populations, supports the theory that these regions are under selective pressure [51]. In addition to the highlighted substitutions, several non-synonymous mutations were identified in the analyzed epitopes. These modifications may be associated with conformational changes in EBNA3C, potentially affecting its efficiency and recognition by molecules such as HLA [52]. Therefore, it can be hypothesized that these mutated epitopes may influence EBNA3C function, stability, recognition, and interactions with the host immune system.

Recombination initiator motifs, such as TGGAG and CCCAG, are commonly found across the EBV genome [53,54]. The CCCAG motif is concentrated within predicted recombinant regions of EBV, suggesting its role in initiating recombination or serving as a breakpoint, as discussed in previous studies [35,53]. Recent studies have shown differences between distinct haplotypes of the virus, particularly the CCCAG motif associated with a higher frequency in EBV-positive patients [54]. Consistent with our results, we found differences in motif abundance between EBV1 and EBV2, with EBV2 having a higher frequency of CCCAG, which may reflect distinct recombination patterns and adaptive strategies between the genotypes. These findings contribute to understanding how motif distribution influences EBNA3C variability. The CCCAG motif is significantly concentrated within predicted recombinant regions of EBV, suggesting its role in initiating recombination or serving as a breakpoint, as discussed in previous studies [35,53].

The analysis of the EBNA3C NLS (NLS-1, NLS-2, and NLS-3) revealed a high degree of conservation across EBV-1 isolates (>99% identity). Conversely, EBV-2 exhibited non-synonymous substitutions in all NLS. Notably, the NLS-2 of EBV-2, where the K418T substitution replaces a positively charged lysine with a neutral threonine, removes the basic residue critical for nuclear transport. This non-synonymous substitution disrupts a key basic residue, which is critical for nuclear transport. Consistent with observations in simian virus 40 (SV40), where comparable mutations in the T-antigen NLS abolished nuclear localization [55]. This implies that EBV-2 NLS-2 may be non-functional, raising questions about compensatory nuclear import mechanisms in this genotype. In NLS-3 EBV-2 isolates, the substitution R1018S, replacing arginine (basic) with serine (polar uncharged), was universally harbored. While this preserves partial polarity, the loss of positive charges likely reduces binding affinity to importin-α, a key mediator of nuclear transport in herpes simplex virus [56]. Notably, such substitutions are absent in EBV-1, underscoring a conserved functional imperative for cationic residues in its NLS motifs. These findings align with broader patterns in herpesvirus evolution, where non-essential motifs in latent proteins tolerate drift, while core functional motifs remain under purifying selection [57,58]. The retention of divergent NLS sequences in EBV-2 may reflect relaxed constraints due to alternative nuclear shuttling mechanisms or lineage-specific adaptations to distinct host–cell environments [59,60].

The RBP-Jk binding domain of EBNA3C harbors four evolutionarily conserved residues (209-TFGC-212) critical for mediating its interaction with the transcriptional repressor RBP-Jk in vivo [61]. Despite substantial divergence across the broader domain (82% sequence dissimilarity between EBV-1 and EBV-2), the TFGC motif remains conserved, underscoring stringent purifying selection to preserve this interaction interface. This conservation likely reflects its non-redundant role in displacing EBNA2 from RBP-Jk, a mechanism essential for EBV-driven B-cell immortalization [62,63,64]. This pattern mirrors observations in other herpesviruses: baboon LCV maintains ~35% EBNA3C N-terminal conservation, including the TFGC motif, despite peripheral divergence [62]. Therapeutic disruption of this interface, for instance, via peptides mimicking the TFGC motif, could competitively inhibit EBNA3C sequestration of RBP-Jk, thereby restoring EBNA2-mediated tumor-suppressive signaling [65,66]. The identification of recombination regions in key functional regions of EBNA3C, such as the NLS, suggests that these structural variations may have functional implications for viral biology. This finding provides insights into potential impacts of mutation and recombination on EBNA3 based on bioinformatics analyses; further experimental analysis is necessary to confirm these findings and their potential contribution to viral pathogenesis.

The analysis of EBNA3C functional motif variability revealed promising opportunities for therapeutic development. The high conservation of NLS-1 in both EBV genotypes suggests that peptide-based strategies could be employed to block EBNA3C nuclear translocation, similar to the approach proposed by Levin et al. (2009) [67]. This work demonstrated that a synthetic peptide derived from the NLS sequence of human immunodeficiency virus 1 (HIV-1) integrase (IN) blocks the protein’s nuclear import. Another interesting result corresponds to the RBP-Jk binding site, in which the amino acids critical for the establishment of LCLs remain completely conserved. This preservation, analogous to that observed in NLS-1, may represent a viable target for competitive inhibition via synthetic peptides.

Targeting the degradation of viral oncoproteins has emerged as a promising therapeutic strategy. In this context, Proteolysis Targeting Chimera (PROTAC) has gained attention. It consists of a small synthetic molecule designed to bind an E3 ubiquitin ligase to a specific target protein, leading to subsequent degradation by the proteasome [68]. This strategy demonstrates efficacy in degrading the human papillomavirus E6 oncoprotein [69]. According to the Sugiokto and Li (2025) study, the EBNA1 synthetic inhibitor could induce EBNA1 degradation through ubiquitination [70]. Taken together, current evidence supports EBNA3C as a therapeutic target through PROTAC-mediated degradation.

## 4. Materials and Methods

### 4.1. EBNA3C Recovery

We performed sequence similarity searches on July 10, 2025, with the Basic Local Alignment Search Tool BLASTn https://blast.ncbi.nlm.nih.gov/Blast.cgi (accessed on 10 July 2025) using the EBNA3C sequence of EBV-1 prototype (NC_007605) from 86083 to 89132 nucleotide (nt) and of EBV-2 prototype (NC_009334) from 86654 to 89934 nt. Sequences of both EBV types were aligned with Mafft http://mafft.cbrc.jp/alignment/server (accessed on 11 July 2025) [71] and manually edited using Ugene v50 [72]. Sequences that contained more than 5% of unresolved nucleotides (Ns) and had less than 75% of similarity with the query were removed from the analysis, along with samples from established cell lines. The metadata of each isolate was annotated in the header of the sequences, including health status, country, and year of collection. A final dataset of 988 sequences was obtained for the study (see Supplementary Table S1). The EBNA3C sequence includes the BERF3 and BERF4 exons and the intronic region between them, which were analyzed to assess sequence variation (nucleotide and amino acid) and recombination regions.

### 4.2. Annotation of EBNA3C Motifs

The EBNA3C protein motifs annotation was performed employing a Uniprot-based search (Supplementary Table S6), using NC_007605 and NC_009334 as queries for EBV-1 and EBV-2, respectively. The key residues of the protein were identified according to the literature: bZIP [14], RBP-Jk [61], Nm23-H1 [25], and NLS (1, 2, and 3) [13]. The epitopes analyzed in this study were described according to the literature [36,73,74].

### 4.3. Recombination Analysis

The recombination analyses of EBNA3C were performed with two distinct software programs, Recombination Detection Program (RPD v5.61) [75] and Genealogies Unbiased by Recombinations in Nucleotide Sequences (Gubbins v2.4.1) [76] to identify the recombination regions based on phylogenetic discrepancies. With EBNA3C recombination regions defined, recombination-inducing motifs for EBV were analyzed as previously described [35,53]. Recombination-inducing motifs were categorized as inside or outside these regions, and occurrences were counted for both viral types. ClonalFrameML v1.13 [77] was employed to assess the impact of recombination and the frequency of recombination regions vs. point mutations. Various methods were applied in RDP5 to detect potential recombination regions. The recombinant regions were considered significant if they were detected by at least 5 of 7 detection methods (RDP, GENECONV, MaxChi, Chimera, SisScan, 3Seq) [78,79,80,81,82,83,84] and if the *p*-value < 0.05 after applying the Bonferroni correction. We used Gubbins to detect recombination regions based on phylogenetic discrepancies.

### 4.4. Phylogenetic Reconstructions and Identification of Population Groups

A phylogenetic reconstruction was performed with the EBNA3C dataset using PhyML 3.0 http://www.atgc-montpellier.fr/phymll/ (accessed on 12 July 2025), applying the Akaike information criterion (AIC) selection criterion and aLRT SH-like fast likelihood-based method. The best-fitting model selected was the GTR + R. This approach was used to compare the reconstruction of the unfiltered and filtered sequences to assess the impact of recombination. The phylogenetic trees were visualized by iTOL https://itol.embl.de/ (accessed on 12 July 2025) [85]. To determine the phylopopulations, we performed a Bayesian analysis of population structure according to the RhierBAPS v1.01 [86] software with the recombinant regions identified with Gubbins and then the intronic regions removed; the run was defined at two levels of hierarchy with 20 initial clusters. The comparison of the tree topologies obtained before and after the filtering process was measured using Robinson–Foulds Distance [87], Matching Split Distance [88], Branch Score Distance [89], and Path Difference [90].

### 4.5. Statistical Analysis

To assess associations between amino acid mutations and categorical metadata (clinical status and country of origin), a custom Python script was implemented. For each variant detected at the selected positions, a presence/absence matrix was constructed and compared between the categories defined in Supplementary Table S1, separately. Associations were assessed using Pearson’s chi-square test. Multiple testing was corrected using the Benjamini–Hochberg false discovery rate (FDR) method and the Bonferroni correction. Associations were considered statistically significant at an adjusted *p*-value ≤ 0.05. All statistical analyses and data visualizations were performed in Python (v3.12).

## 5. Conclusions

This study demonstrated that variability in the EBNA3C gene is pivotal due to recombination regions, which contributed five times more to substitutions than mutation. and contribute significantly to the emergence of divergent motifs within functionally important regions such as Nm23-H1, RBP-Jκ, and nuclear localization signals. Despite this extensive variability, the EBNA3C gene considered alone does not have sufficient phylogenetic signal to discriminate monophyletic phylopopulations. EBNA3C diversity is more closely associated with geographic origin than with specific disease phenotypes. These results provide new insights into how molecular evolution could shape the virus cycle. Further experimental studies are warranted to elucidate the functional consequences of the identified variants and to better understand their potential impact on viral pathogenesis and oncogenesis.

## Figures and Tables

**Table 1:** Overview of amino acid variation in BERF3 and BERF4. The table compares the amino acid substitution patterns of BERF3 and BERF4, highlighting their overall frequency and the diseases related with this pattern. Moreover, it provides the geographic distribution of these disease-related patterns and deletions. No amino acid variation patterns were found in BERF3 of the EBV-2 genotype. Amino acid patterns are represented as wild-type position patterns (XposY), while deletions are denoted by their range start…end (pos1…pos2). The most significant results are highlighted in bold.

Region	Amino Acid Substitution Pattern	Frequency (%)	Disease (%)	Country (%)
BERF3 EBV-1	T107I	4.6	Healthy (88.1)	China (97.3)
				Taiwan (2.7)
	T104A + T107V	19.0	CAEBV (28.5%)	Japan (100.0)
			Healthy (21.2)	China (89.5)
				Taiwan (5.3)
				UK (5.3)
			NPC (14.0)	China (96.0)
				Indonesia (4.0)
			NKTCL (8.4)	Japan (60.0)
				China (13.3)
				Indonesia (13.3)
				Singapore (13.3)
	L45F + Q60P + T104A + T107V	1.8	IM (37.5)	USA (100.0)
			PTLD (25.0)	UK (100.0)
			BL (18.8)	Africa (33.3)
				North Africa (33.3)
				USA (33.3)
	R11I + N21D + R44G + Y51D	16.3	NPC (24.8)	China (100.0)
			CAEBV (24.2)	Japan (97.2)
				USA (2.8)
			Healthy (15.4)	China (91.3)
				UK (8.7)
			GC (13.4)	China (65.0)
				South Korea (25.0)
				Japan (5.0)
				Korea (5.0)
	R11I + N21D + R44G + Y51D + T107I	30.4	NPC (62.6)	China (99.4)
				Indonesia (0.6)
			Healthy (25.2)	China (98.6)
				Taiwan (1.4)
			pLELC (7.6)	China (100.0)
BERF4 EBV-1	I141V + Q213H + E336D + I348L + R656G + E701Q	27.0	NPC (64.8)	China (99.4)
				Indonesia (0.6)
			Healthy (22.7)	China (98.2)
				UK (1.8)
			pLELC (7.3)	China (100.0)
	I348L + L669P + S690P + E701Q + H831Y + C915W	3.8	Healthy (88.6)	China (96.8)
				Taiwan (3.2)
			NKTCL (11.4)	China (75.0)
				Singapore (25.0)
	I141V + Q213H + E336D + I348L + L669P + E701Q + C915W	2.4	CAEBV (50.0)	Japan (100.0)
	I141V + Q213H + E336D + I348L + L669P + E701Q + C915W + A978V	1.5	GC (42.9)	South Korea (50.0)
				China (16.7)
				Japan (16.7)
				Korea (16.7)
			CAEBV (35.7)	Japan (100.0)
	A162V + G357V + G655S + T677M + A683V + E701Q + Q740P + Q744R + P753Q + L866S	4.7	Healthy (46.5)	China (90.0)
				Taiwan (5.0)
				UK (5.0)
			CAEBV (18.6)	Japan (100.0)
	I141V + A162V + T188A + A215G + T356M + G357V + E701Q + A840S + M904L + A921S	2.4	IM (54.5)	USA (100.0)
			ND (22.7)	UK (80.0)
				Germany (20.0)
	A162V + S557L + G655S + T677M + A683V + E701Q + Q740P + Q744R + P753Q + L866S + K976E	2.5	CAEBV (65.2)	Japan (100.0%)
			IM (17.4)	Japan (50.0)
			NKTCL (13.0)	Japan (100.0)
BERF4 EBV-2	R376S + G472E + V719T + F806V + P829S+ S868L + L881S	11.0	Healthy (62.5)	China (100)
	404…434 + 680…702 + 713…731 + 818…824	13.7	BL (84)	Kenya (100)

**Table 2:** EBNA3C epitope alterations related to medical conditions and geographic distribution. The table details the amino acid variations in eight described epitopes on EBNA3C.

Epitope	Viral Type	Amino Acid Substitutions	Frequency (%)	Clinical Condition (%)	Country (%)
FRKAQIQGL	EBV-1	I348L	55.8	NPC (46.7)	China (100)
				Healthy (25.3)	
		I348M	1.5	BL (85.7)	Kenya (92)
	EBV-2	R344L + I348R	100	BL (27)	Kenya (80)
				Healthy (35)	China (80)
KEHVIQNAF	EBV-1	E336D	46.8	NPC (51.6)	China (100)
				Healthy (20.3)	
		H337Q	1.5	BL (85.7)	Kenya (92)
	EBV-2	H337Q + N341K	100	BL (27)	Kenya (80)
				Healthy (35)	China (80)
LRGKWQRRYR	EBV-2	Y257F	100	BL (27)	Kenya (80)
				Healthy (35)	China (80)
RRIYDLIEL	EBV-1	R259K	3.2	Healthy (20.7)	United Kingdom (100)
				IM (20.7)	United States (100)
				PTLD (13.8)	United Kingdom (100)
		Y261F	2.4	NPC (50)	Indonesia (91)
	EBV-2	Y261F	100	BL (27)	Kenya (80)
				Healthy (35)	China (80)
QPRAPIRPI	EBV-2	Q761P + R763P	19	Healthy (14)	Kenya (100)
				BL (20)	

Burkitt lymphoma (BL), Infectious Mononucleosis (IM), Conserved, showing no changes (-).

## Data Availability

All data obtained in this study are detailed in the text and in the Supplementary Materials.

## Supplementary Materials

The following supporting information can be downloaded at https://www.mdpi.com/article/10.3390/ijms27073054/s1.
