# Supplementary material for: Exploring EBNA3C Genetic Variability and Recombination in Epstein–Barr Virus-Associated Cancers
Source: Int J Mol Sci. 2026 Mar 27;27(7):3054. doi: 10.3390/ijms27073054 (PMC13072985; doi:10.3390/ijms27073054)
Supplement: Supplementary file 1 [file ijms-27-03054-s001.zip › ijms-3743150-supplementary.pdf]

Supplementary Material

Supplementary Table S1. List of sequences analyzed in this study with its metadata and the population assigned in this study.

Accession Number	Isolate	Health Status	Country	Year	Population
AB850654	HN15 Hunan	NPC	China	2012	pop1
AJ507799	B95-8.Raji	IM	USA	1984	pop1
AP015015	SNU-719	GC	Japan	2016	pop3
AP015016	YCCEL1	GC	Japan	2015	pop6
AP019012	KAI3 cell	LCL	Japan	2013	pop6
AP019013	SNK1 cell	LCL	Japan	2013	pop4
AP019014	SNK10 cell	LCL	Japan	2013	pop4
AP019015	SNT13 cell	LCL	Japan	2013	pop6
AP019016	SNT15 cell	LCL	Japan	2013	pop6
AP019017	SNT16 cell	LCL	Japan	2013	pop6
AP019018	SNT8 cell	LCL	Japan	2013	pop4
AP019019	UPN1002 PBMC	DLBCL	Japan	2013	pop6
AP019020	UPN1003 PBMC	DLBCL	Japan	2013	pop6
AP019025	UPN102 PBMC	CAEBV	Japan	2013	pop6
AP019026	UPN104 PBMC	CAEBV	Japan	2013	pop6
AP019027	UPN105 PBMC	CAEBV	Japan	2013	pop4
AP019028	UPN105 tumor	CAEBV	Japan	2013	pop4
AP019029	UPN106 PBMC	CAEBV	Japan	2013	pop4
AP019030	UPN107 PBMC	CAEBV	Japan	2013	pop4
AP019032	UPN108 tumor	CAEBV	Japan	2013	pop4
AP019033	UPN109 PBMC	CAEBV	Japan	2013	pop6
AP019034	UPN110 PBMC	CAEBV	Japan	2013	pop6
AP019035	UPN1101 PBMC	NKTCL	Japan	2013	pop6
AP019036	UPN1102 PBMC	NKTCL	Japan	2013	pop4
AP019037	UPN1104 PBMC	NKTCL	Japan	2013	pop6
AP019038	UPN1106 PBMC	NKTCL	Japan	2013	pop4
AP019039	UPN111 PBMC	CAEBV	Japan	2013	pop6
AP019040	UPN1110 PBMC	NKTCL	Japan	2013	pop4
AP019042	UPN1113 PBMC	NKTCL	Japan	2013	pop4

AP019044	UPN112 PBMC	CAEBV	Japan	2013	pop4
AP019045	UPN1120 PBMC	PBMC	Japan	2013	pop6
AP019047	UPN113 PBMC	CAEBV	Japan	2013	pop6
AP019048	UPN114 PBMC	CAEBV	Japan	2013	pop4
AP019049	UPN114 tumor	CAEBV	Japan	2013	pop4
AP019051	UPN1201 PBMC	NKTCL	Japan	2013	pop4
AP019052	UPN1202 PBMC	NKTCL	Japan	2013	pop6
AP019053	UPN123 tumor	CAEBV	Japan	2013	pop4
AP019054	UPN125 PBMC	CAEBV	Japan	2013	pop6
AP019055	UPN128 PBMC	CAEBV	Japan	2013	pop6
AP019056	UPN128 tumor	CAEBV	Japan	2013	pop6
AP019059	UPN130 tumor	CAEBV	Japan	2013	pop6
AP019060	UPN132 PBMC	CAEBV	Japan	2013	pop4
AP019061	UPN132 tumor	CAEBV	Japan	2013	pop4
AP019062	UPN134 PBMC	PBMC	Japan	2013	pop4
AP019064	UPN136 PBMC	CAEBV	Japan	2013	pop4
AP019065	UPN136 tumor	CAEBV	Japan	2013	pop4
AP019067	UPN138 tumor	CAEBV	Japan	2013	pop4
AP019070	UPN140 tumor	CAEBV	Japan	2013	pop4
AP019071	UPN142 gdT-1	CAEBV	Japan	2013	pop6
AP019072	UPN142 gdT-2	CAEBV	Japan	2013	pop6
AP019073	UPN142 PBMC	CAEBV	Japan	2013	pop6
AP019076	UPN1756 PBMC	NKTCL	Japan	2013	pop4
AP019077	UPN1757 PBMC	NKTCL	Japan	2013	pop6
AP019078	UPN1758 PBMC	NKTCL	Japan	2013	pop6
AP019079	UPN1789 PBMC	NKTCL	Japan	2013	pop4
AP019080	UPN18 PBMC	PTLD	Japan	2013	pop4
AP019081	UPN1802 PBMC	NKTCL	Japan	2013	pop6
AP019082	UPN1813 PBMC	NKTCL	Japan	2013	pop6
AP019083	UPN1833 tissue	LE	Japan	2013	pop4
AP019086	UPN1901 tissue	LM	Japan	2013	pop6
AP019087	UPN1926 PBMC	NKTCL	Japan	2013	pop4
AP019093	UPN2329 PBMC BD	DLBCL	Japan	2013	pop6
AP019096	UPN251 PBMC	CAEBV	Japan	2013	pop4
AP019097	UPN252 PBMC	CAEBV	Japan	2013	pop6
AP019098	UPN253 PBMC	CAEBV	Japan	2013	pop6
AP019099	UPN254 PBMC	CAEBV	Japan	2013	pop6
AP019100	UPN255 PBMC	CAEBV	Japan	2013	pop6

AP019102	UPN257 PBMC	CAEBV	Japan	2013	pop4
AP019103	UPN258 PBMC	CAEBV	Japan	2013	pop6
AP019104	UPN259 PBMC	CAEBV	Japan	2013	pop4
AP019106	UPN260 PBMC	CAEBV	Japan	2013	pop6
AP019107	UPN261 PBMC	CAEBV	Japan	2013	pop6
AP019108	UPN262 PBMC	CAEBV	Japan	2013	pop4
AP019109	UPN263 PBMC	CAEBV	Japan	2013	pop6
AP019111	UPN265	PBMC	Japan	2013	pop4
AP019112	UPN2654 PBMC	DLBCL	Japan	2013	pop4
AP019113	UPN266 PBMC	CAEBV	Japan	2013	pop6
AP019114	UPN267 PBMC	CAEBV	Japan	2013	pop4
AP019118	UPN301 CD56+	CAEBV	Japan	2013	pop4
AP019119	UPN303 CD56+	CAEBV	Japan	2013	pop6
AP019120	UPN306 CD56+	CAEBV	Japan	2013	pop6
AP019121	UPN3065 PBMC	NKTCL	Japan	2013	pop6
AP019122	UPN307 CD56+	CAEBV	Japan	2013	pop6
AP019123	UPN310 CD56+	CAEBV	Japan	2013	pop6
AP019124	UPN311 CD56+	CAEBV	Japan	2013	pop4
AP019125	UPN3198 PBMC	NKTCL	Japan	2013	pop4
AP019126	UPN322 CD3+	CAEBV	Japan	2013	pop6
AP019127	UPN322 CD56+	CAEBV	Japan	2013	pop6
AP019128	UPN326 PBMC	CAEBV	Japan	2013	pop6
AP019129	UPN3300 PBMC	NKTCL	Japan	2013	pop6
AP019130	UPN3448 PBMC	DLBCL	Japan	2013	pop6
AP019131	UPN3621 PBMC	NKTCL	Japan	2013	pop6
AP019132	UPN365 CD3+	CAEBV	Japan	2013	pop6
AP019133	UPN365 CD56+	CAEBV	Japan	2013	pop6
AP019134	UPN366 CD3+	CAEBV	Japan	2013	pop6
AP019135	UPN366 CD56+	CAEBV	Japan	2013	pop4
AP019136	UPN3707 PBMC	DLBCL	Japan	2013	pop4
AP019139	UPN3755 tissue	LE	Japan	2013	pop6
AP019140	UPN404 CD3+	CAEBV	Japan	2013	pop4
AP019141	UPN404 CD56+	CAEBV	Japan	2013	pop4
AP019142	UPN404 PBMC	CAEBV	Japan	2013	pop4
AP019143	UPN405 CD3+	CAEBV	Japan	2013	pop6
AP019144	UPN405 CD56+	CAEBV	Japan	2013	pop6
AP019145	UPN412 CD3+	CAEBV	Japan	2013	pop6
AP019147	UPN412 PBMC	CAEBV	Japan	2013	pop6

AP019148	UPN4137 tissue	LM	Japan	2013	pop4
AP019150	UPN417 CD56+	CAEBV	Japan	2013	pop4
AP019151	UPN417 PBMC	CAEBV	Japan	2013	pop4
AP019155	UPN4314 PBMC	DLBCL	Japan	2013	pop6
AP019157	UPN470 PBMC	CAEBV	Japan	2013	pop6
AP019159	UPN486 PBMC	CAEBV	Japan	2013	pop6
AP019160	UPN494 CD3+	CAEBV	Japan	2013	pop6
AP019161	UPN494 CD56+	CAEBV	Japan	2013	pop6
AP019162	UPN495 PBMC	CAEBV	Japan	2013	pop4
AP019163	UPN497 PBMC	CAEBV	Japan	2013	pop6
AP019164	UPN498 PBMC	CAEBV	Japan	2013	pop4
AP019166	UPN5087 PBMC	DLBCL	Japan	2013	pop4
AP019168	UPN519 PBMC	CAEBV	Japan	2013	pop6
AP019169	UPN520 PBMC	CAEBV	Japan	2013	pop4
AP019170	UPN539 PBMC	CAEBV	Japan	2013	pop6
AP019171	UPN554 PBMC	CAEBV	Japan	2013	pop6
AP019172	UPN5575 PBMC	NKTCL	Japan	2013	pop4
AP019173	UPN563 CD19+	CAEBV	Japan	2013	pop6
AP019174	UPN563 CD3+	CAEBV	Japan	2013	pop6
AP019175	UPN563 CD56+	CAEBV	Japan	2013	pop6
AP019176	UPN571 CD3+	CAEBV	Japan	2013	pop6
AP019177	UPN571 CD56+	CAEBV	Japan	2013	pop6
AP019179	UPN582 PBMC	CAEBV	Japan	2013	pop4
AP019180	UPN584 PBMC	CAEBV	Japan	2013	pop4
AP019181	UPN874 PBMC	NKTCL	Japan	2013	pop4
AP019182	UPN88 CD56+-1	CAEBV	Japan	2013	pop4
AP019183	UPN88 CD56+-3	CAEBV	Japan	2013	pop4
AP019184	UPN93 gdT-2	CAEBV	Japan	2013	pop6
AP019185	UPN97 PBMC	CAEBV	Japan	2013	pop6
AP019186	UPN97 tumor	CAEBV	Japan	2013	pop6
AP019187	UPN98 PBMC	CAEBV	Japan	2013	pop4
AP019188	UPN99 PBMC	CAEBV	Japan	2013	pop6
AY961628	GD1	Healthy	China	1998	pop6
DQ279927	AG876	BL	Ghana	2009	pop5
HQ020558	GD2	NPC	China	2009	pop4
HZ437644	JP 2015530369-A/11	ND	USA	2012	pop5
JQ009376	HKNPC1	NPC	China	2008	pop4
KC207813	Akata	BL	Japan	2012	pop4

KC207814	Mutu	BL	Kenya	2012	pop2
KC440851	K4123-Mi	LCL	USA	2012	pop1
KC440852	K4123-MiEBV	LCL	USA	2012	pop10
KC617875	C666-1	NPC	China	1998	pop4
KF373730	M81	NPC	China	1970	pop4
KF992564	HKNPC2	NPC	China	2008	pop4
KF992565	HKNPC3	NPC	China	2008	pop4
KF992566	HKNPC4	NPC	China	2008	pop4
KF992567	HKNPC5	NPC	China	2008	pop4
KF992568	HKNPC6	NPC	China	2008	pop7
KF992569	HKNPC7	NPC	China	2008	pop7
KF992570	HKNPC8	NPC	China	2008	pop4
KF992571	HKNPC9	NPC	China	2008	pop4
KJ411974	C666-1	NPC	China	1998	pop3
KP735248	GC1	GC	SK	2010	pop3
KP968257	CCH	BL	Brazil	1980	pop1
KP968258	MP	BL	Brazil	1980	pop1
KP968259	SCL	BL	Brazil	1980	pop1
KP968261	HU11393	BL	Ghana	1980	pop2
KP968262	H018436D	BL	Ghana	1976	pop1
KP968263	H058015C	BL	Ghana	1990	pop1
KP968264	H002213	BL	Ghana	1981	pop1
KR063343	CV-ARG	BL	Argentina	1980	pop1
KR063344	RPF	BL	Brazil	1980	pop8
KR063345	FNR	BL	Brazil	1980	pop2
KT001103	SG	BL	Argentina	1981	pop1
KT254013	EBVaGC3	GC	China	2014	pop4
KT273942	EBVaGC1	GC	China	2013	pop6
KT273943	EBVaGC2	GC	China	2013	pop4
KT273945	EBVaGC5	GC	China	2009	pop6
KT273946	EBVaGC6	GC	China	2009	pop4
KT273947	EBVaGC7	GC	China	2009	pop4
KT273948	EBVaGC8	GC	China	2010	pop3
KT273949	EBVaGC9	GC	China	2010	pop4
KT823506	LC1	LC	China	2014	pop4
KT823507	LC2	LC	China	2014	pop6
KT823508	LC3	LC	China	2014	pop4
KT823509	LC4	LC	China	2014	pop3

KX125050	GC-EBV1	GC	China	2011	pop4
KX125051	GC-EBV2	GC	China	2012	pop6
KX125052	SNU-719	GC	SK	2008	pop3
KX125053	YCCEL1	GC	SK	2010	pop6
KX674064	GDGC1	GC	China	2011	pop4
KX674065	GDGC2	GC	China	2012	pop6
KX674066	SNU-719	GC	SK	2008	pop3
KX674067	YCCEL1	GC	SK	2010	pop6
LC573550	sLCL-T2.27 TS	sLCL	Japan	2020	pop5
LC573551	sLCL-T3.27 TS	sLCL	Japan	2020	pop6
LC573552	sLCL-T12.18 TS	sLCL	Japan	2020	pop5
LC573553	sLCL-T12.20 TS	sLCL	Japan	2020	pop6
LC573554	sLCL-T12.22 TS	sLCL	Japan	2020	pop6
LC573555	sLCL-T12.11 TS	sLCL	Japan	2020	pop3
LC573556	sLCL-T1.12 TS	sLCL	Japan	2020	pop6
LR812977	eBL CL-03	sBLCL	Kenya	2009	pop2
LR812979	eBL HC-0004	Healthy	Kenya	2009	pop5
LR812981	HC-0002	Healthy	Kenya	2009	pop5
LR812983	eBL HC-0005	Healthy	Kenya	2009	pop5
LR812984	HC-0007	Healthy	Kenya	2009	pop1
LR812985	HC-0006	Healthy	Kenya	2009	pop5
LR812987	HC-0009	Healthy	Kenya	2009	pop2
LR812990	HC-0012	Healthy	Kenya	2009	pop5
LR812994	HC-0018	Healthy	Kenya	2009	pop5
LR812995	HC-0015	Healthy	Kenya	2009	pop1
LR812995	HC-0015	Healthy	Kenya	2009	pop1
LR812996	HC-0016	Healthy	Kenya	2009	pop5
LR812997	HC-0019	Healthy	Kenya	2009	pop5
LR812998	HC-0028	Healthy	Kenya	2009	pop5
LR813000	HC-0026	Healthy	Kenya	2009	pop5
LR813001	HC-0027	Healthy	Kenya	2009	pop5
LR813002	HC-0029	Healthy	Kenya	2009	pop2
LR813004	HC-0021	Healthy	Kenya	2009	pop2
LR813009	HC-0034	Healthy	Kenya	2009	pop5
LR813011	HC-0030	Healthy	Kenya	2009	pop1
LR813014	HC-0037	Healthy	Kenya	2009	pop1
LR813018	eBL-Tumor-0011	BL	Kenya	2009	pop8
LR813019	eBL-Tumor-0012	BL	Kenya	2009	pop5

LR813020	eBL-Tumor-0002	BL	Kenya	2009	pop1
LR813021	eBL-Tumor-0008	BL	Kenya	2009	pop5
LR813023	eBL-Tumor-0001	BL	Kenya	2009	pop8
LR813024	eBL-Tumor-0007	BL	Kenya	2009	pop8
LR813026	eBL-Tumor-0006	BL	Kenya	2009	pop1
LR813028	eBL-Tumor-0005	BL	Kenya	2009	pop2
LR813031	eBL-Tumor-0015	BL	Kenya	2009	pop8
LR813032	eBL-Tumor-0014	BL	Kenya	2009	pop2
LR813033	eBL-Tumor-0013	BL	Kenya	2009	pop2
LR813034	eBL-Tumor-0020	BL	Kenya	2009	pop5
LR813035	eBL-Tumor-0018	BL	Kenya	2009	pop8
LR813038	eBL-Tumor-0022	BL	Kenya	2009	pop1
LR813040	eBL-Tumor-0024	BL	Kenya	2009	pop5
LR813042	eBL-Tumor-0017	BL	Kenya	2009	pop5
LR813043	eBL-Tumor-0029	BL	Kenya	2009	pop8
LR813044	eBL-Tumor-0031	BL	Kenya	2009	pop1
LR813045	eBL-Tumor-0028	BL	Kenya	2009	pop5
LR813047	eBL-Tumor-0026	BL	Kenya	2009	pop5
LR813048	eBL-Tumor-0030	BL	Kenya	2009	pop2
LR813050	eBL-Tumor-0032	BL	Kenya	2009	pop8
LR813051	eBL-Tumor-0036	BL	Kenya	2009	pop5
LR813052	eBL-Tumor-0033	BL	Kenya	2009	pop5
LR813053	eBL-Tumor-0035	BL	Kenya	2009	pop5
LR813054	eBL-Tumor-0037	BL	Kenya	2009	pop8
LR813055	eBL-Tumor-0034	BL	Kenya	2009	pop1
LR813057	eBL-Plasma-0047	BL	Kenya	2009	pop2
LR813058	eBL-Plasma-0046	BL	Kenya	2009	pop2
LR813059	eBL-Plasma-0045	BL	Kenya	2009	pop2
LR813060	eBL-Plasma-0042	BL	Kenya	2009	pop1
LR813061	eBL-Plasma-0043	BL	Kenya	2009	pop5
LR813063	eBL-Tumor-0040	BL	Kenya	2009	pop5
LR813064	eBL-Tumor-0041	BL	Kenya	2009	pop8
LR813065	eBL-Plasma-0044	BL	Kenya	2009	pop2
LR813069	eBL-Plasma-0054	BL	Kenya	2009	pop5
LR813070	eBL-Plasma-0053	BL	Kenya	2009	pop2
LR813071	eBL-Plasma-0052	BL	Kenya	2009	pop1
LR813072	eBL-Plasma-0051	BL	Kenya	2009	pop2
LR813073	eBL-Plasma-0035	BL	Kenya	2009	pop5

LR813075	eBL-Plasma-0055	BL	Kenya	2009	pop8
LR813079	eBL-Plasma-0040	BL	Kenya	2009	pop5
LR813143	eBL-Plasma-0036	BL	Kenya	2009	pop5
LR994476	NPC-12T	NPC	China	2022	pop4
LR994477	NPC-14T	NPC	China	2022	pop4
LR994478	NPC-13T	NPC	China	2022	pop4
LR994479	NPC-15T	NPC	China	2022	pop4
LR994480	NPC-16T	NPC	China	2022	pop4
LR994481	NPC-19T	NPC	China	2022	pop4
LR994483	NPC-2T	NPC	China	2022	pop4
LR994484	NPC-3T	NPC	China	2022	pop4
LR994486	NPC-18T	NPC	China	2022	pop4
LR994487	NPC-8T	NPC	China	2022	pop4
LR994488	NPC-11T	NPC	China	2022	pop4
LR994489	NPC-20T	NPC	China	2022	pop4
LR994490	NPC-10T	NPC	China	2022	pop4
LR994491	NPC-4T	NPC	China	2022	pop4
LR994492	NPC-1T	NPC	China	2022	pop4
LR994493	NPC-9T	NPC	China	2022	pop4
LR994494	NPC-17T	NPC	China	2022	pop4
LR994495	NPC-6T	NPC	China	2022	pop4
LR994501	NPC-22T	NPC	China	2022	pop4
LR994502	NPC-26T	NPC	China	2022	pop4
LR994503	NPC-29T	NPC	China	2022	pop4
LR994507	NPC-23T	NPC	China	2022	pop4
LR994508	NPC-21T	NPC	China	2022	pop6
LR994510	NPC-40T	NPC	China	2022	pop4
LR994513	NPC-24T	NPC	China	2022	pop4
LR994516	NPC-38T	NPC	China	2022	pop4
LR994518	NPC-37T	NPC	China	2022	pop4
LR994519	NPC-25T	NPC	China	2022	pop4
LR994520	NPC-30T	NPC	China	2022	pop4
LR994521	NPC-41T	NPC	China	2022	pop4
LR994522	NPC-42T	NPC	China	2022	pop5
LR994523	NPC-51T	NPC	China	2022	pop4
LR994526	NPC-58T	NPC	China	2022	pop4
LR994527	NPC-43T	NPC	China	2022	pop5
LR994529	NPC-45T	NPC	China	2022	pop4

LR994532	NPC-59T	NPC	China	2022	pop4
LR994533	NPC-55T	NPC	China	2022	pop4
LR994534	NPC-62T	NPC	China	2022	pop4
LR994535	NPC-49T	NPC	China	2022	pop6
LR994536	NPC-47T	NPC	China	2022	pop4
LR994537	NPC-50T	NPC	China	2022	pop4
LR994541	NPC-61T	NPC	China	2022	pop4
LS992239	EBV6	ND	UK	2012	pop10
LS992240	P4-T1	PTLD	UK	2003	pop10
LS992242	EBV7	ND	UK	2012	pop2
LS992243	EBV9	ND	UK	2012	pop2
LS992244	EBV13	ND	UK	2012	pop8
LS992245	EBV15	ND	UK	2013	pop2
LS992257	P2-T1	PTLD	UK	2012	pop1
LS992258	P1-T1	PTLD	UK	2015	pop10
LS992260	P3-2670	IM	Japan	2018	pop6
LS992262	P4-2274	CEBVD	Japan	2008	pop6
LS992263	P5-1294	IM	Japan	2018	pop6
LS992264	P11-871	IM	Japan	2018	pop3
LS992265	P9-2631	IM	Japan	2018	pop6
LS992266	P8-414	IM	Japan	2018	pop6
MB445486	JP 2018138581-A/11	ND	USA	2018	pop5
MF547454	E1583 OWv1	IM	USA	2013	pop2
MF547456	E1583 OWv7	IM	USA	2014	pop2
MF547457	E1587 BCv1	IM	USA	2013	pop10
MF547458	E1587 OWv1	IM	USA	2013	pop1
MF547459	E1587 BCv7	IM	USA	2014	pop1
MF547460	E1587 OWv7	IM	USA	2014	pop1
MF547461	E1536 BCv1	IM	USA	2012	pop2
MF547462	E1536 OWv1	IM	USA	2012	pop2
MF547463	E1563 BCv1	IM	USA	2013	pop2
MF547464	E1536 OWv7	IM	USA	2013	pop2
MF547465	E1536 BCv7	IM	USA	2013	pop1
MF547466	E1548 BCv1	IM	USA	2013	pop2
MF547467	E1548 OWv1	IM	USA	2013	pop1
MF547468	E1548 BCv7	IM	USA	2013	pop1
MF547469	E1548 OWv7	IM	USA	2013	pop1
MF547470	E1563 OWv1	IM	USA	2013	pop2

MF547471	E1563 OWv7	IM	USA	2014	pop2
MF547472	E1563 BCV7	IM	USA	2014	pop1
MF547473	E1590 BCv1	IM	USA	2014	pop10
MF547474	E1590 OWv1	IM	USA	2014	pop10
MF547475	E1590 BCv7	IM	USA	2014	pop1
MF547476	E1590 OWv7	IM	USA	2014	pop1
MF547477	E1492 BCv1	IM	USA	2010	pop2
MF547478	E1492 OWv1	IM	USA	2010	pop2
MF547479	E1492 BCv7	IM	USA	2011	pop2
MF547480	E1492 OWv7	IM	USA	2011	pop1
MF547481	E1503 BCv1	IM	USA	2010	pop2
MF547482	E1503 OWv1	IM	USA	2010	pop2
MF547483	E1503 BCv7	IM	USA	2011	pop2
MF547484	E1503 OWv7	IM	USA	2011	pop2
MF547485	E1578 BCv1	IM	USA	2013	pop10
MF547486	E1578 BCv7	IM	USA	2014	pop1
MF547487	E1578 OWv7	IM	USA	2014	pop1
MF547488	E1578 OWv1	IM	USA	2013	pop10
MF547489	E1577 BCv1	IM	USA	2013	pop10
MF547490	E1577 OWv1	IM	USA	2013	pop10
MF547491	E1577 OWv7	IM	USA	2017	pop10
MF547492	E1577 BCv7	IM	USA	2017	pop1
MG021305	YCCEL1-GC1	GC	China	2014	pop6
MG021306	YCCEL1-GC2	GC	Poland	2013	pop6
MG021307	Akata-GC1	GC	SK	2013	pop4
MG021308	Mutu-GC1	GC	Poland	2013	pop2
MG021309	Mutu-GC2	GC	USA	2013	pop1
MG021310	Mutu-GC3	GC	Poland	2013	pop10
MG021311	Mutu-GC4	GC	Ukraine	2013	pop10
MG021312	AG876-GC1	GC	SK	2017	pop5
MG021313	EBVaGC8-1	GC	SK	2013	pop3
MG021314	EBVaGC8-2	GC	China	2014	pop3
MG021315	EBVaGC8-3	GC	China	2014	pop3
MG021316	HKNPC6-GC1	GC	Vietnam	2013	pop3
MG021317	EBVaGC5-1	GC	SK	2013	pop6
MG298823	AH Saliva 8192	Healthy	Taiwan	2015	pop6
MG298824	AH Saliva 8471	Healthy	Taiwan	2015	pop9
MG298825	AH Saliva 8489	Healthy	Taiwan	2015	pop7

MG298826	AH Saliva 9077	Healthy	Taiwan	2015	pop6
MG298827	AH Saliva 9316	Healthy	Taiwan	2015	pop5
MG298828	DF Tonsil T47	BL	Argentina	2015	pop1
MG298829	DF Tonsil T49	BL	Argentina	2015	pop1
MG298830	GK Akuba	BL	Kenya	2015	pop8
MG298831	GK BL16	BL	NorthAfrica	2015	pop5
MG298832	GK BL18	BL	NorthAfrica	2015	pop10
MG298833	GK BL36	BL	NorthAfrica	2015	pop1
MG298834	GK BL42	BL	NorthAfrica	2015	pop1
MG298835	GK BL44	BL	NorthAfrica	2015	pop1
MG298836	GK BL60	BL	NorthAfrica	2015	pop2
MG298837	GK BL67	BL	France	2015	pop2
MG298838	GK BL72	BL	NorthAfrica	2015	pop1
MG298839	GK Farage	BL	USA	2015	pop10
MG298840	GK LY47	BL	East Africa	2015	pop1
MG298841	GK LY65	BL	East Africa	2015	pop2
MG298842	GK PUT	BL	Africa	2015	pop5
MG298844	IMS Saliva 10	Healthy	UK	2015	pop1
MG298845	IMS Saliva 12	Healthy	UK	2015	pop6
MG298846	IMS Saliva 120	Healthy	UK	2015	pop4
MG298847	IMS Saliva 162	Healthy	UK	2015	pop1
MG298848	IMS Saliva 170	Healthy	UK	2015	pop2
MG298850	IMS Saliva 193	Healthy	UK	2015	pop9
MG298851	IMS Saliva 204	Healthy	UK	2015	pop9
MG298853	IMS Saliva 231	Healthy	UK	2015	pop9
MG298854	IMS Saliva 243	Healthy	UK	2015	pop4
MG298855	IMS Saliva 248	Healthy	UK	2015	pop2
MG298856	IMS Saliva 250	Healthy	UK	2015	pop2
MG298857	IMS Saliva 31	Healthy	UK	2015	pop2
MG298858	IMS Saliva 49	Healthy	UK	2015	pop6
MG298859	IMS Saliva 52	Healthy	UK	2015	pop9
MG298860	IMS Saliva 6	Healthy	UK	2015	pop2
MG298861	IMS Saliva 70	Healthy	UK	2015	pop10
MG298862	IMS Saliva 71	Healthy	UK	2015	pop9
MG298863	IMS Saliva 9	Healthy	UK	2015	pop10
MG298864	JC 002	CAEBV	USA	2015	pop2
MG298865	JC 023	CAEBV	USA	2015	pop4

MG298866	JC 030 18	CAEBV	USA	2015	pop2
MG298867	JC 030 29	CAEBV	USA	2015	pop2
MG298868	JC 037	CAEBV	USA	2015	pop2
MG298869	JC 040	CAEBV	USA	2015	pop9
MG298870	JC V005	CAEBV	USA	2015	pop2
MG298871	JC V006	CAEBV	USA	2015	pop2
MG298872	JM IK sLCL	LCL	Indonesia	2015	pop2
MG298873	JM IN sLCL	LCL	Indonesia	2015	pop9
MG298874	JM MU sLCL	LCL	Indonesia	2015	pop9
MG298875	JM SU sLCL	LCL	Indonesia	2015	pop9
MG298876	JM NKTLY 218.1	NKTCL	Indonesia	2015	pop9
MG298877	JM NKTLY 96.1	NKTCL	Indonesia	2015	pop6
MG298878	JM NKTLY 98.1	NKTCL	Indonesia	2015	pop6
MG298879	JM biop 162 NPC	NPC	Indonesia	2015	pop9
MG298880	JM biop 238 NPC	NPC	Indonesia	2015	pop9
MG298881	JM biop 27 NPC	NPC	Indonesia	2015	pop1
MG298882	JM biop 51 NPC	NPC	Indonesia	2015	pop9
MG298883	JM bru 178 NPC	NPC	Indonesia	2015	pop9
MG298884	JM bru 238 NPC	NPC	Indonesia	2015	pop9
MG298885	JM bru 377 NPC	NPC	Indonesia	2015	pop9
MG298886	JM bru 38 NPC	NPC	Indonesia	2015	pop9
MG298887	JM bru 51 NPC	NPC	Indonesia	2015	pop9
MG298889	JM bru L2 NPC	NPC	Indonesia	2015	pop9
MG298890	JM bru L29 NPC	NPC	Indonesia	2015	pop9
MG298891	JM bru L3 NPC	NPC	Indonesia	2015	pop9
MG298892	JM bru L37 NPC	NPC	Indonesia	2015	pop6
MG298893	JM bru L4 NPC	NPC	Indonesia	2015	pop5
MG298894	JM bru L41 NPC	NPC	Indonesia	2015	pop9
MG298895	JM bru L42 NPC	NPC	Indonesia	2015	pop9
MG298896	JM bru L47 NPC	NPC	Indonesia	2015	pop4
MG298897	JM bru L5 NPC	NPC	Indonesia	2015	pop9
MG298898	JM bru L7 NPC	NPC	Indonesia	2015	pop5
MG298899	JM Saliva 18	BL	East Africa	2015	pop2
MG298901	JM Saliva 33	BL	East Africa	2015	pop1
MG298902	JM Saliva 5	BL	East Africa	2015	pop2
MG298903	JWBL121B	BL	Africa	2015	pop2
MG298904	JWBL17A	BL	Africa	2015	pop10
MG298905	JWBL43B	BL	Africa	2015	pop1

MG298906	M81 gDNA	LCL	China	2015	pop4
MG298907	RK H12 sLCL	LCL	PNG	2015	pop9
MG298908	RK H16 sLCL	LCL	PNG	2015	pop9
MG298909	RK H35 sLCL	LCL	PNG	2015	pop9
MG298910	RK L12 sLCL	LCL	PNG	2015	pop9
MG298911	RK L19	LCL	UK	2015	pop5
MG298912	RK L2 sLCL	LCL	PNG	2015	pop9
MG298913	RK L24 sLCL	LCL	PNG	2015	pop9
MG298914	RK L3 sLCL	LCL	PNG	2015	pop5
MG298915	RK L4 sLCL	LCL	PNG	2015	pop5
MG298916	RK L5 sLCL	LCL	PNG	2015	pop9
MG298917	DF Tonsil T156 BWA	LCL	Argentina	2015	pop1
MG298919	IMS Saliva 155 BWA	Healthy	UK	2015	pop2
MG298920	IMS Saliva 187 BWA	Healthy	UK	2015	pop2
MG298921	IMS Saliva 216 BWA	Healthy	UK	2015	pop10
MG298922	IMS Saliva 5 BWA	Healthy	UK	2015	pop1
MG298923	IMS Saliva 81 BWA	Healthy	UK	2015	pop5
MG298925	JC 027 BWA	CAEBV	USA	2015	pop2
MG298926	JC 039 BWA	CAEBV	USA	2015	pop1
MG298928	JC VID41 BWA	CAEBV	USA	2015	pop1
MH101958	NKTCL-SC02	NKTCL	China	2016	pop9
MH101959	NKTCL-SC03	NKTCL	China	2016	pop4
MH101960	NKTCL-SC04	NKTCL	China	2016	pop3
MH101961	NKTCL-SC05	NKTCL	China	2016	pop9
MH101962	NKTCL-SC06	NKTCL	China	2016	pop4
MH101963	NKTCL-SC07	NKTCL	China	2016	pop6
MH101964	NKTCL-SC08	NKTCL	China	2016	pop6
MH101965	NKTCL-SC09	NKTCL	China	2016	pop4
MH101966	NKTCL-SC10	NKTCL	China	2016	pop7
MH101967	NKTCL-SC11	NKTCL	China	2015	pop7
MH101968	NKTCL-SC12	NKTCL	China	2015	pop9
MH101969	NKTCL-SC13	NKTCL	China	2015	pop9
MH101970	NKTCL-SC14	NKTCL	China	2016	pop9
MH101971	NKTCL-SC15	NKTCL	China	2018	pop9
MH144212	NKTCL-SG01	NKTCL	Singapore	2018	pop6
MH144213	NKTCL-SG02	NKTCL	Singapore	2018	pop9
MH144215	NKTCL-SG04	NKTCL	Singapore	2018	pop6
MH144216	NKTCL-SG05	NKTCL	Singapore	2019	pop5

MH144218	NKTCL-SG07	NKTCL	Singapore	2018	pop7
MH144219	NKTCL-SG08	NKTCL	Singapore	2018	pop2
MH144221	NKTCL-SG10	NKTCL	Singapore	2018	pop9
MH144223	NKTCL-SG12	NKTCL	Singapore	2018	pop6
MH590370	HKHD1	Healthy	China	2018	pop4
MH590371	HKHD2	Healthy	China	2018	pop6
MH590372	HKHD3	Healthy	China	2018	pop9
MH590373	HKHD4	Healthy	China	2018	pop4
MH590374	HKHD5	Healthy	China	2018	pop6
MH590375	HKHD6	Healthy	China	2018	pop4
MH590376	HKHD7	Healthy	China	2018	pop7
MH590378	HKHD9	Healthy	China	2018	pop7
MH590379	HKHD10	Healthy	China	2018	pop4
MH590380	HKHD11	Healthy	China	2018	pop6
MH590381	HKHD12	Healthy	China	2018	pop5
MH590382	HKHD13	Healthy	China	2018	pop7
MH590383	HKHD14	Healthy	China	2018	pop4
MH590384	HKHD15	Healthy	China	2018	pop6
MH590385	HKHD16	Healthy	China	2018	pop6
MH590386	HKHD17	Healthy	China	2018	pop4
MH590387	HKHD18	Healthy	China	2018	pop2
MH590388	HKHD19	Healthy	China	2018	pop7
MH590389	HKHD20	Healthy	China	2018	pop4
MH590390	HKHD21	Healthy	China	2018	pop6
MH590391	HKHD22	Healthy	China	2018	pop6
MH590392	HKHD23	Healthy	China	2018	pop6
MH590393	HKHD24	Healthy	China	2018	pop9
MH590394	HKHD25	Healthy	China	2018	pop9
MH590395	HKHD26	Healthy	China	2018	pop4
MH590396	HKHD27	Healthy	China	2018	pop7
MH590397	HKHD28	Healthy	China	2018	pop7
MH590399	HKHD30	Healthy	China	2018	pop3
MH590400	HKHD31	Healthy	China	2018	pop7
MH590401	HKHD32	Healthy	China	2018	pop7
MH590402	HKHD33	Healthy	China	2018	pop9
MH590403	HKHD34	Healthy	China	2018	pop4
MH590404	HKHD35	Healthy	China	2018	pop4
MH590405	HKHD36	Healthy	China	2018	pop4

MH590406	HKHD37	Healthy	China	2018	pop4
MH590407	HKHD38	Healthy	China	2018	pop4
MH590408	HKHD39	Healthy	China	2018	pop4
MH590409	HKHD40	Healthy	China	2018	pop4
MH590410	HKHD41	Healthy	China	2018	pop5
MH590411	HKHD42	Healthy	China	2018	pop7
MH590412	HKHD43	Healthy	China	2018	pop3
MH590413	HKHD44	Healthy	China	2018	pop7
MH590414	HKHD45	Healthy	China	2018	pop4
MH590416	HKHD47	Healthy	China	2018	pop4
MH590417	HKHD48	Healthy	China	2018	pop5
MH590418	HKHD49	Healthy	China	2018	pop6
MH590419	HKHD50	Healthy	China	2018	pop7
MH590420	HKHD51	Healthy	China	2018	pop9
MH590421	HKHD52	Healthy	China	2018	pop5
MH590422	HKHD53	Healthy	China	2018	pop5
MH590423	HKHD54	Healthy	China	2018	pop4
MH590424	HKHD55	Healthy	China	2018	pop7
MH590425	HKHD56	Healthy	China	2018	pop7
MH590426	HKHD57	Healthy	China	2018	pop4
MH590427	HKHD58	Healthy	China	2018	pop4
MH590428	HKHD59	Healthy	China	2018	pop9
MH590429	HKHD60	Healthy	China	2018	pop7
MH590430	HKHD61	Healthy	China	2018	pop7
MH590431	HKHD62	Healthy	China	2018	pop6
MH590432	HKHD63	Healthy	China	2018	pop4
MH590433	HKHD64	Healthy	China	2018	pop4
MH590434	HKHD65	ND	China	2018	pop4
MH590435	HKHD66	Healthy	China	2018	pop7
MH590436	HKHD67	Healthy	China	2018	pop5
MH590437	HKHD68	Healthy	China	2018	pop9
MH590438	HKHD69	Healthy	China	2018	pop7
MH590439	HKHD70	Healthy	China	2018	pop7
MH590440	HKHD71	Healthy	China	2018	pop7
MH590441	HKHD72	Healthy	China	2018	pop5
MH590442	HKHD73	Healthy	China	2018	pop4
MH590443	HKHD74	Healthy	China	2018	pop7
MH590444	HKHD75	Healthy	China	2018	pop7

MH590445	HKHD76	Healthy	China	2018	pop4
MH590446	HKHD77	Healthy	China	2018	pop5
MH590447	HKHD78	Healthy	China	2018	pop4
MH590448	HKHD79	Healthy	China	2018	pop5
MH590449	HKHD80	Healthy	China	2018	pop7
MH590450	HKHD81	Healthy	China	2018	pop6
MH590451	HKHD82	Healthy	China	2018	pop7
MH590452	HKHD83	Healthy	China	2018	pop5
MH590453	HKHD84	Healthy	China	2018	pop3
MH590454	HKHD85	Healthy	China	2018	pop4
MH590455	HKHD86	Healthy	China	2018	pop7
MH590456	HKHD87	Healthy	China	2018	pop4
MH590457	HKHD88	Healthy	China	2018	pop4
MH590458	HKHD89	Healthy	China	2018	pop7
MH590459	HKHD90	Healthy	China	2018	pop9
MH590460	HKHD91	Healthy	China	2018	pop4
MH590461	HKHD92	Healthy	China	2018	pop4
MH590462	HKHD93	Healthy	China	2018	pop6
MH590463	HKHD94	Healthy	China	2018	pop4
MH590464	HKHD95	Healthy	China	2018	pop6
MH590465	HKHD96	Healthy	China	2018	pop6
MH590466	HKHD97	Healthy	China	2018	pop5
MH590467	HKHD98	Healthy	China	2018	pop5
MH590468	HKHD99	Healthy	China	2018	pop7
MH590469	HKHD100	Healthy	China	2018	pop4
MH590470	HKHD101	Healthy	China	2018	pop4
MH590471	HKHD102	Healthy	China	2018	pop4
MH590472	HKHD103	Healthy	China	2018	pop6
MH590473	HKHD104	Healthy	China	2018	pop5
MH590474	HKHD105	Healthy	China	2018	pop4
MH590475	HKHD106	Healthy	China	2018	pop6
MH590476	HKHD107	Healthy	China	2018	pop6
MH590477	HKHD108	Healthy	China	2018	pop7
MH590478	HKHD109	Healthy	China	2018	pop6
MH590479	HKHD110	Healthy	China	2018	pop4
MH590480	HKHD111	Healthy	China	2018	pop4
MH590481	HKHD112	Healthy	China	2018	pop7
MH590483	HKHD114	Healthy	China	2018	pop7

MH590484	HKHD115	Healthy	China	2018	pop3
MH590485	HKHD116	Healthy	China	2018	pop4
MH590486	HKHD117	Healthy	China	2018	pop6
MH590487	HKHD118	Healthy	China	2018	pop6
MH590488	HKHD119	Healthy	China	2018	pop5
MH590489	HKHD120	Healthy	China	2018	pop6
MH590491	HKHD122	Healthy	China	2018	pop9
MH590492	HKHD123	Healthy	China	2018	pop5
MH590493	HKHD124	Healthy	China	2018	pop7
MH590494	HKHD125	Healthy	China	2018	pop6
MH590495	HKHD126	Healthy	China	2018	pop9
MH590496	HKHD127	Healthy	China	2018	pop6
MH590497	HKHD128	Healthy	China	2018	pop3
MH590498	HKHD129	Healthy	China	2018	pop4
MH590500	HKHD131	Healthy	China	2018	pop4
MH590501	HKHD132	Healthy	China	2018	pop7
MH590503	HKHD134	Healthy	China	2018	pop5
MH590504	HKHD135	Healthy	China	2018	pop4
MH590505	HKHD136	Healthy	China	2018	pop5
MH590506	HKHD137	Healthy	China	2018	pop6
MH590507	HKHD138	Healthy	China	2018	pop9
MH590508	HKHD139	Healthy	China	2018	pop5
MH590509	HKHD140	Healthy	China	2018	pop3
MH590510	HKHD141	Healthy	China	2018	pop5
MH590511	HKHD142	Healthy	China	2018	pop9
MH590512	HKNPC1	NPC	China	2018	pop4
MH590513	HKNPC2	NPC	China	2018	pop4
MH590514	HKNPC3	NPC	China	2018	pop4
MH590515	HKNPC4	NPC	China	2018	pop4
MH590516	HKNPC5	NPC	China	2018	pop4
MH590517	HKNPC6	NPC	China	2018	pop7
MH590518	HKNPC7	NPC	China	2018	pop7
MH590519	HKNPC8	NPC	China	2018	pop4
MH590520	HKNPC9	NPC	China	2018	pop4
MH590521	HKNPC10	NPC	China	2018	pop4
MH590522	HKNPC11	NPC	China	2018	pop4
MH590523	HKNPC12	NPC	China	2018	pop4
MH590524	HKNPC13	NPC	China	2018	pop4

MH590525	HKNPC14	NPC	China	2018	pop4
MH590526	HKNPC15	NPC	China	2018	pop4
MH590527	HKNPC16	NPC	China	2018	pop4
MH590528	HKNPC17	NPC	China	2018	pop4
MH590529	HKNPC18	NPC	China	2018	pop4
MH590530	HKNPC19	NPC	China	2018	pop7
MH590531	HKNPC20	NPC	China	2018	pop4
MH590532	HKNPC21	NPC	China	2018	pop4
MH590533	HKNPC22	NPC	China	2018	pop4
MH590534	HKNPC23	NPC	China	2018	pop4
MH590535	HKNPC24	NPC	China	2018	pop4
MH590536	HKNPC25	NPC	China	2018	pop4
MH590537	HKNPC26	NPC	China	2018	pop7
MH590538	HKNPC27	NPC	China	2018	pop4
MH590539	HKNPC28	NPC	China	2018	pop7
MH590540	HKNPC29	NPC	China	2018	pop4
MH590541	HKNPC30	NPC	China	2018	pop4
MH590542	HKNPC31	NPC	China	2018	pop4
MH590543	HKNPC32	NPC	China	2018	pop4
MH590544	HKNPC33	NPC	China	2018	pop4
MH590545	HKNPC34	NPC	China	2018	pop3
MH590546	HKNPC35	NPC	China	2018	pop6
MH590547	HKNPC36	NPC	China	2018	pop6
MH590548	HKNPC37	NPC	China	2018	pop4
MH590549	HKNPC38	NPC	China	2018	pop6
MH590550	HKNPC39	NPC	China	2018	pop9
MH590551	HKNPC40	NPC	China	2018	pop3
MH590552	HKNPC41	NPC	China	2018	pop4
MH590553	HKNPC42	NPC	China	2018	pop4
MH590554	HKNPC43	NPC	China	2018	pop3
MH590555	HKNPC44	NPC	China	2018	pop4
MH590556	HKNPC45	NPC	China	2018	pop5
MH590557	HKNPC46	NPC	China	2018	pop4
MH590558	HKNPC47	NPC	China	2018	pop7
MH590559	HKNPC48	NPC	China	2018	pop4
MH590560	HKNPC49	NPC	China	2018	pop4
MH590561	HKNPC50	NPC	China	2018	pop4
MH590562	HKNPC51	NPC	China	2018	pop4

MH590563	HKNPC52	NPC	China	2018	pop7
MH590564	HKNPC53	NPC	China	2018	pop4
MH590565	HKNPC54	NPC	China	2018	pop4
MH590566	HKNPC55	NPC	China	2018	pop4
MH590567	HKNPC56	NPC	China	2018	pop7
MH590568	HKNPC57	NPC	China	2018	pop4
MH590569	HKNPC58	NPC	China	2018	pop4
MH590570	HKNPC59	NPC	China	2018	pop4
MH590571	HKNPC60	NPC	China	2018	pop3
MH590572	HKNPC61	NPC	China	2018	pop7
MH590573	HKNPC62	NPC	China	2018	pop6
MH590574	B95-8 IM-cellline	ND	China	2018	pop1
MH590575	C6661	NPC	China	2018	pop3
MH590576	Jijoye	BL	USA	2019	pop5
MH590577	M81	NPC	China	2018	pop4
MH590578	NPC43	NPC	China	2018	pop4
MH590579	SNU719	GC	Korea	2018	pop3
MH837516	AIL13	AIL	France	2018	pop2
MH837520	ARL2	ND	France	2018	pop9
MH837521	CTCL1	AIL	France	2018	pop10
MH837522	DLBCL2	DLBCL	France	2018	pop9
MH837524	NKTL2	NKTCL	France	2018	pop2
MH837525	PTBL1	PTBL	France	2018	pop2
MH837526	PTBL2	PTBL	France	2018	pop2
MH883755	ebv6	ND	UK	2018	pop10
MH883756	ebv7	ND	UK	2018	pop2
MH883758	ebv9	ND	UK	2018	pop2
MH883759	ebv13	ND	UK	2018	pop8
MH883761	ebv15	ND	UK	2018	pop2
MH883773	P1-T1	PTLD	UK	2018	pop10
MH883775	P2-T1	PTLD	UK	2018	pop1
MH883776	P3-2670	IM	UK	2018	pop6
MH883777	P3-T1	PTLD	UK	2018	pop2
MH883778	P4-2274	ND	UK	2018	pop6
MH883779	P4-T1	PTLD	UK	2018	pop10
MH883780	P5-1294	IM	UK	2018	pop6
MH883783	P8-414	IM	UK	2018	pop6
MH883784	P9-2631	IM	UK	2018	pop6

MH883785	P11-871	IM	UK	2018	pop3
MK540241	BLT001	BL	China	2013	pop3
MK540242	BLT002	BL	China	2013	pop3
MK540243	C666	NPC	China	1998	pop3
MK540244	GCT001	GC	China	2013	pop3
MK540245	GCT002	GC	China	2013	pop6
MK540246	GCT003	GC	China	2013	pop3
MK540247	GCT004	GC	China	2013	pop6
MK540248	GCT005	GC	China	2013	pop6
MK540249	GCT006	GC	China	2013	pop6
MK540250	GCT007	GC	China	2013	pop3
MK540251	GCT009	GC	China	2013	pop6
MK540252	GCT010	GC	China	2013	pop3
MK540253	GCT011	GC	China	2013	pop3
MK540254	GCT012	GC	China	2013	pop6
MK540255	GCT013	GC	China	2013	pop6
MK540256	GCT014	GC	China	2013	pop3
MK540257	HLT001	HL	China	2011	pop6
MK540258	HLT002	HL	China	2011	pop6
MK540259	HLT005	HL	China	2012	pop3
MK540260	HLT006	HL	China	2012	pop3
MK540261	HLT007	HL	China	2012	pop3
MK540262	HLT010	HL	China	2013	pop7
MK540263	HLT011	HL	China	2013	pop3
MK540264	HS001	Healthy	China	2013	pop3
MK540265	HS003	Healthy	China	2013	pop3
MK540266	HS007	Healthy	China	2010	pop3
MK540267	HS008	Healthy	China	2011	pop3
MK540268	HS009	Healthy	China	2011	pop3
MK540269	HS011	Healthy	China	2011	pop6
MK540270	HS012	Healthy	China	2011	pop6
MK540271	HS013	Healthy	China	2012	pop3
MK540272	HS014	Healthy	China	2012	pop3
MK540273	HS015	Healthy	China	2012	pop6
MK540274	HS016	Healthy	China	2012	pop3
MK540275	HS018	Healthy	China	2010	pop3
MK540276	HS019	Healthy	China	2010	pop3
MK540277	HS020	Healthy	China	2010	pop3

MK540278	HS021	Healthy	China	2012	pop3
MK540279	HS023	Healthy	China	2012	pop6
MK540280	HS024	Healthy	China	2012	pop6
MK540281	HS025	Healthy	China	2013	pop3
MK540282	HS027	Healthy	China	2011	pop3
MK540283	HS029	Healthy	China	2011	pop3
MK540284	HS032	Healthy	China	2012	pop7
MK540285	HS033	Healthy	China	2012	pop7
MK540286	HS034	Healthy	China	2013	pop3
MK540287	HS035	Healthy	China	2014	pop7
MK540288	HS036	Healthy	China	2014	pop7
MK540289	HS037	Healthy	China	2014	pop3
MK540290	HS038	Healthy	China	2014	pop3
MK540291	HS039	Healthy	China	2014	pop3
MK540292	HS041	Healthy	China	2014	pop3
MK540293	HS045	Healthy	China	2013	pop3
MK540294	HS048	Healthy	China	2013	pop3
MK540295	HS050	Healthy	China	2013	pop3
MK540296	HS051	Healthy	China	2013	pop3
MK540297	HS052	Healthy	China	2013	pop3
MK540298	HS053	Healthy	China	2013	pop3
MK540299	HS054	Healthy	China	2013	pop3
MK540300	HS057	Healthy	China	2013	pop6
MK540301	NHS002	Healthy	China	2013	pop3
MK540302	NHS004	Healthy	China	2013	pop3
MK540303	NKLT002	NKTCL	China	2012	pop3
MK540304	NKLT003-2	NKTCL	China	2013	pop3
MK540305	NKLT004	NKTCL	China	2013	pop3
MK540306	NKLT006	NKTCL	China	2013	pop3
MK540307	NKLT007	NKTCL	China	2013	pop7
MK540308	NNPCT001	NPC	China	2013	pop6
MK540309	NNPCT002	NPC	China	2013	pop3
MK540310	NNPCT003	NPC	China	2013	pop6
MK540311	NNPCT004	NPC	China	2013	pop3
MK540312	NNPCT005	NPC	China	2013	pop6
MK540313	NPCP001	NPC	China	2013	pop3
MK540314	NPCS001	NPC	China	2011	pop3
MK540315	NPCS002	NPC	China	2011	pop3

MK540316	NPCS003-2	NPC	China	2011	pop3
MK540317	NPCS005	NPC	China	2011	pop3
MK540318	NPCS006	NPC	China	2011	pop3
MK540319	NPCS007	NPC	China	2011	pop3
MK540320	NPCS008	NPC	China	2011	pop3
MK540321	NPCS009	NPC	China	2011	pop3
MK540322	NPCS010	NPC	China	2011	pop6
MK540323	NPCS011	NPC	China	2011	pop3
MK540324	NPCS012	NPC	China	2011	pop3
MK540325	NPCS013	NPC	China	2011	pop3
MK540326	NPCS014	NPC	China	2011	pop3
MK540327	NPCS016	NPC	China	2011	pop3
MK540328	NPCS017	NPC	China	2011	pop3
MK540329	NPCS018	NPC	China	2011	pop3
MK540330	NPCS019	NPC	China	2011	pop3
MK540331	NPCS021	NPC	China	2011	pop3
MK540332	NPCS022	NPC	China	2011	pop3
MK540333	NPCS023	NPC	China	2011	pop6
MK540334	NPCS024	NPC	China	2011	pop3
MK540335	NPCS025	NPC	China	2011	pop3
MK540336	NPCS026	NPC	China	2012	pop3
MK540337	NPCS027	NPC	China	2012	pop3
MK540338	NPCS028	NPC	China	2012	pop3
MK540339	NPCS029	NPC	China	2012	pop3
MK540340	NPCS030	NPC	China	2012	pop3
MK540341	NPCS031	NPC	China	2012	pop6
MK540342	NPCS033	NPC	China	2012	pop3
MK540343	NPCS034	NPC	China	2012	pop3
MK540344	NPCS035	NPC	China	2012	pop3
MK540345	NPCS038	NPC	China	2012	pop3
MK540346	NPCS039	NPC	China	2012	pop3
MK540347	NPCS040	NPC	China	2012	pop3
MK540348	NPCS042	NPC	China	2012	pop3
MK540349	NPCS044	NPC	China	2012	pop3
MK540350	NPCS045	NPC	China	2013	pop3
MK540351	NPCS046	NPC	China	2013	pop3
MK540352	NPCS047	NPC	China	2013	pop3
MK540353	NPCS048	NPC	China	2013	pop3

MK540354	NPCS049	NPC	China	2013	pop3
MK540355	NPCS050	NPC	China	2012	pop3
MK540356	NPCS051	NPC	China	2013	pop3
MK540357	NPCS052	NPC	China	2013	pop6
MK540358	NPCS054	NPC	China	2013	pop3
MK540359	NPCT001	NPC	China	2013	pop3
MK540360	NPCT002	NPC	China	2013	pop3
MK540361	NPCT003	NPC	China	2013	pop3
MK540362	NPCT004	NPC	China	2012	pop3
MK540363	NPCT005	NPC	China	2013	pop3
MK540364	NPCT006	NPC	China	2013	pop3
MK540365	NPCT007	NPC	China	2013	pop3
MK540366	NPCT008	NPC	China	2013	pop3
MK540367	NPCT009	NPC	China	2013	pop6
MK540368	NPCT010	NPC	China	2013	pop3
MK540369	NPCT011	NPC	China	2013	pop3
MK540370	NPCT012	NPC	China	2013	pop3
MK540371	NPCT013	NPC	China	2015	pop3
MK540372	NPCT014	NPC	China	2013	pop3
MK540373	NPCT015	NPC	China	2013	pop6
MK540374	NPCT017	NPC	China	2013	pop3
MK540375	NPCT018	NPC	China	2013	pop3
MK540376	NPCT019	NPC	China	2013	pop3
MK540377	NPCT020-2	NPC	China	2013	pop6
MK540378	NPCT021	NPC	China	2013	pop5
MK540379	NPCT022	NPC	China	2013	pop3
MK540380	NPCT023	NPC	China	2013	pop3
MK540381	NPCT024	NPC	China	2013	pop3
MK540382	NPCT025	NPC	China	2013	pop3
MK540383	NPCT027	NPC	China	2013	pop3
MK540384	NPCT028-2	NPC	China	2013	pop3
MK540385	NPCT029	NPC	China	2013	pop3
MK540386	NPCT031	NPC	China	2013	pop3
MK540387	NPCT032	NPC	China	2013	pop3
MK540388	NPCT033	NPC	China	2013	pop3
MK540389	NPCT035	NPC	China	2013	pop3
MK540390	NPCT036	NPC	China	2013	pop3
MK540391	NPCT037	NPC	China	2013	pop3

MK540392	NPCT038	NPC	China	2013	pop3
MK540393	NPCT039	NPC	China	2013	pop6
MK540394	NPCT040	NPC	China	2013	pop3
MK540395	NPCT041	NPC	China	2013	pop3
MK540396	NPCT042	NPC	China	2013	pop6
MK540397	NPCT043	NPC	China	2013	pop3
MK540398	NPCT045	NPC	China	2013	pop3
MK540399	NPCT046	NPC	China	2013	pop3
MK540400	NPCT047	NPC	China	2013	pop3
MK540401	NPCT048	NPC	China	2013	pop6
MK540402	NPCT049	NPC	China	2013	pop5
MK540403	NPCT050	NPC	China	2013	pop3
MK540404	NPCT052	NPC	China	2013	pop3
MK540405	NPCT053	NPC	China	2013	pop3
MK540406	NPCT054	NPC	China	2013	pop3
MK540407	NPCT054M	NPC	China	2013	pop3
MK540408	NPCT055	NPC	China	2013	pop3
MK540409	NPCT055M	NPC	China	2013	pop3
MK540410	NPCT056	NPC	China	2013	pop3
MK540411	NPCT056M	NPC	China	2013	pop3
MK540412	NPCT057	NPC	China	2013	pop3
MK540413	NPCT057M	NPC	China	2013	pop3
MK540414	NPCT058	NPC	China	2013	pop3
MK540415	NPCT058M	NPC	China	2013	pop3
MK540416	NPCT059	NPC	China	2013	pop3
MK540417	NPCT060	NPC	China	2013	pop3
MK540418	NPCT061	NPC	China	2013	pop3
MK540419	NPCT062	NPC	China	2013	pop3
MK540420	NPCT063	NPC	China	2013	pop3
MK540421	NPCT064	NPC	China	2013	pop3
MK540422	NPCT065	NPC	China	2013	pop6
MK540423	NPCT066	NPC	China	2013	pop3
MK540424	NPCT067	NPC	China	2013	pop3
MK540425	NPCT068	NPC	China	2013	pop3
MK540426	NPCT069	NPC	China	2013	pop3
MK540427	NPCT070	NPC	China	2013	pop3
MK540428	NPCT071	NPC	China	2013	pop3
MK540429	NPCT072	NPC	China	2013	pop6

MK540430	NPCT073	NPC	China	2013	pop3
MK540431	NPCT074	NPC	China	2014	pop3
MK540432	NPCT074S	NPC	China	2014	pop3
MK540433	NPCT075	NPC	China	2013	pop3
MK540434	NPCT076	NPC	China	2013	pop3
MK540435	NPCT077	NPC	China	2013	pop3
MK540436	NPCT078	NPC	China	2013	pop3
MK540437	NPCT080	NPC	China	2013	pop3
MK540438	NPCT081	NPC	China	2013	pop3
MK540439	NPCT082	NPC	China	2013	pop3
MK540440	NPCT083	NPC	China	2013	pop3
MK540441	NPCT084	NPC	China	2013	pop3
MK540442	NPCT085	NPC	China	2013	pop3
MK540443	NPCT086	NPC	China	2013	pop3
MK540444	NPCT087	NPC	China	2013	pop6
MK540445	NPCT088	NPC	China	2013	pop3
MK540446	NPCT089	NPC	China	2013	pop6
MK540447	NPCT090	NPC	China	2013	pop7
MK540448	NPCT091	NPC	China	2013	pop3
MK540449	NPCT092	NPC	China	2013	pop3
MK540450	NPCT093	NPC	China	2013	pop3
MK540451	NPCT094	NPC	China	2013	pop3
MK540452	NPCT096	NPC	China	2013	pop3
MK540453	NPCT098	NPC	China	2013	pop3
MK540454	NPCT099	NPC	China	2013	pop3
MK540455	NPCT100	NPC	China	2013	pop3
MK540456	NPCT101	NPC	China	2013	pop3
MK540457	NPCT102	NPC	China	2013	pop3
MK540458	NPCT103	NPC	China	2013	pop3
MK540459	NPCT104	NPC	China	2013	pop3
MK540460	NPCT105	NPC	China	2013	pop3
MK540461	NPCT106	NPC	China	2013	pop6
MK540462	NPCT107	NPC	China	2013	pop3
MK540463	NPCT108	NPC	China	2013	pop3
MK540464	NPCT109	NPC	China	2013	pop6
MK540465	NPCT110	NPC	China	2013	pop3
MK540466	NPCT111	NPC	China	2013	pop6
MK540467	NPCT112	NPC	China	2013	pop3

MK540468	NPCT113	NPC	China	2013	pop3
MK540469	NPCT114	NPC	China	2013	pop3
MK540470	NPCT115	NPC	China	2013	pop3
MK973061	IM-3 Heidelberg	IM	Germany	2014	pop2
MK973062	rMSHJ	ND	Germany	2015	pop2
MT648642	Necker-1	TCD	France	2020	pop5
MT648644	Necker-3	TCD	France	2020	pop2
MT648645	Necker-4	TCD	France	2020	pop2
MT648646	Necker-5	TCD	France	2020	pop2
MT648648	Necker-7	TCD	France	2020	pop1
MT648649	Necker-8	TCD	France	2020	pop2
MT648650	Necker-9	TCD	France	2020	pop1
MT648651	Necker-10	TCD	France	2020	pop9
MT648652	Necker-11	TCD	France	2020	pop1
MT648653	Necker-12	TCD	France	2020	pop2
MT648654	Necker-13	TCD	France	2020	pop2
MT648655	Necker-14	TCD	France	2020	pop6
MT648657	Necker-16	TCD	France	2020	pop5
MT648658	Necker-17	TCD	France	2020	pop10
MT648659	Necker-18	TCD	France	2020	pop1
MT648660	Necker-19	TCD	France	2020	pop1
MT648661	Necker-20	TCD	France	2020	pop2
MT648661	Necker-21	TCD	France	2020	pop2
MT648662	Necker-21	NKTCL	France	2020	pop10
NC007605	B95-8Raji	ND	USA	2018	pop1
OM022118	H13	Healthy	China	2020	pop6
OM022121	H13	Healthy	China	2020	pop5
OM022122	H17	Healthy	China	2020	pop4
OM022123	H18	Healthy	China	2020	pop6
OM022124	H19	Healthy	China	2020	pop6
OM022125	H1	Healthy	China	2020	pop6
OM022126	H20	Healthy	China	2020	pop4
OM022127	H21	Healthy	China	2020	pop4
OM022128	H22	Healthy	China	2020	pop6
OM022129	H23	Healthy	China	2020	pop5
OM022132	H27	Healthy	China	2020	pop4
OM022133	H28	Healthy	China	2020	pop4
OM022134	H29	Healthy	China	2020	pop7

OM022137	H33	Healthy	China	2020	pop5
OM022142	H39	Healthy	China	2020	pop6
OM022145	H42	Healthy	China	2020	pop4
OM022148	H6	Healthy	China	2020	pop4
OM022151	H6	Healthy	China	2020	pop5
OM022151	P10 spLELC	pLELC	China	2020	pop5
OM022152	P10	pLELC	China	2020	pop9
OM022154	P12 pLELC	pLELC	China	2020	pop4
OM022154	P46 pLELC	pLELC	China	2020	pop4
OM022155	P13 pLELC	pLELC	China	2020	pop4
OM022157	P15 pLELC	pLELC	China	2020	pop4
OM022167	P24 pLELC	pLELC	China	2020	pop3
OM022174	P30 pLELC	pLELC	China	2020	pop6
OM022175	P31 pLELC	pLELC	China	2020	pop6
OM022185	P40 pLELC	pLELC	China	2020	pop6
OM022187	P42 pLELC	pLELC	China	2020	pop4
OM022189	P44	pLELC	China	2020	pop3
OM022190	P45 pLELC	pLELC	China	2020	pop4
OM022191	P46	pLELC	China	2020	pop4
OM022194	P49 pLELC	pLELC	China	2020	pop4
OM022196	P50 pLELC	pLELC	China	2020	pop4
OM022197	P51 pLELC	pLELC	China	2020	pop4
OM022198	P52 pLELC	pLELC	China	2020	pop4
OM022200	P54 pLELC	pLELC	China	2020	pop6
OM022203	P57 pLELC	pLELC	China	2020	pop4
OM022205	P59 pLELC	pLELC	China	2020	pop4
OM022206	P5	pLELC	China	2020	pop5
OM022207	P60 pLELC	pLELC	China	2020	pop4
OM022209	P62 pLELC	pLELC	China	2020	pop6
OM022211	P64 pLELC	pLELC	China	2020	pop4
OM022212	P65 pLELC	pLELC	China	2020	pop4
OM022213	P66 pLELC	pLELC	China	2020	pop6
OM022214	P67 pLELC	pLELC	China	2020	pop4
OM022215	P68 pLELC	pLELC	China	2020	pop4
OM022216	P69 pLELC	pLELC	China	2020	pop4
OM022218	P70 pLELC	pLELC	China	2020	pop6
OM022219	P71 pLELC	pLELC	China	2020	pop4
OM022220	P72 pLELC	pLELC	China	2020	pop4

OM022221	P73 pLELC	pLELC	China	2020	pop6
OM022222	P74 pLELC	pLELC	China	2020	pop4
OM022225	P77 pLELC	pLELC	China	2020	pop4
OM022226	P78	pLELC	China	2020	pop4
OR652420	719	ND	Kenya	2016	pop5
OR652422	725	ND	Kenya	2016	pop5
NC009334	AG876	ND	USA	2018	pop5

Health status abbreviations: Angioimmunoblastic T lymphoma (AIL), AIDS-related lymphoma (ARL), Burkitt Lymphoma (BL), Chronic Active EBV Infection (CAEBV), Diffuse large B-cell Lymphoma (DLBCL), Gastric Cancer (GC), Hodgkin Lymphoma (HL), Infectious Mononucleosis (IM), Lung Carcinoma (LC), Lymphoblastoid Cell-line (LCL), Lymphoepithelioma (LE), Lymphoid Malignancy (LM), Natural Killer T-cell Lymphoma (NKTCL), Nasopharyngeal Carcinoma (NPC), Post-transplant B Lymphoma (PTBL), Posttransplant Lymphoproliferative Disorder (PTLD), Pulmonary Lymphoepithelioma-like Carcinoma (PLELC), Spontaneous Lymphoblastoid Cell-line (sLCL), and Not Determined (ND).

Country abbreviations: United Kingdom (UK), United Statest of America (USA), Papua New Guinea (PNG), and South Korea (SK)

Supplementary Table S2. Recombination results for RDP5 software using all recombination methods.

Event Number	Begin	End	Recombinant Sequence(s)	Parental Sequence	
				First Minor	First Major
1	1582	2907	MK540402<br>MK540378	LR813004	MT648642
8	1890	2148	KR063345	LR813018	MF547471
15~	897	1248	MG298898	MK973061	MT648657
17	1277	2577	MH590475<br>MH590573	MG298824	MH837522
23~	3211	3353	KP968264	MG298898	MG298860
29~	2899	3544	OM022167	MF547486	LR812996
39	1261	2224	MG298853<br>MG298883<br>MG298889	MK540280	MG298851

Event numbers with a ~ symbol mean that the recombination signal might be related to other evolutionary events rather than recombination.

Supplementary Table S3. RDP5 recombination prediction p-Values for each tested method.

Event Number	RDP	GENECONV	Bootscan	Maxchi	Chimaera	SiSscan	3Seq
1	1,05E-21	6,68E-19	5,29E-14	1,28E-21	4,12E-21	4,74E-30	6,49E-42
8	2,41E-09	1,36E-05	ns	5,63E-08	1,14E-07	6,90E-18	1,67E-10
15~	ns	3,50E-14	1,96E-15	4,73E-02	4,72E-02	1,94E-04	1,38E-07
17	1,81E-02	6,90E-03	ns	2,53E-07	1,94E-07	7,14E-14	1,09E-10
23~	1,27E-11	1,14E-09	ns	1,12E-05	1,01E-03	6,85E-04	6,38E-06
29~	9,54E-03	1,18E-03	ns	5,99E-08	1,83E-05	2,29E-07	7,62E-11
39	4,90E-04	2,06E-02	ns	3,81E-04	1,05E-03	1,49E-08	1,79E-07

ns cells mean that p-Values were not significant.

Supplementary Table S4. Protein motifs found in this study and a comparison between EBV1 and EBV2, start and end, are expressed in terms of alignment of EBV 1 reference genome NC_007605 and EBV 2 reference genome NC_009334.

Protein Motif	EBV 1	EBV 2	N° of aa	Start	End
NLS -1	RIRRRRRRR	RIRRRRRRR	9	72	80
NLS -2	KKPRK	RKPRT	5	414	418
NLS -3	PKRPRVE	PKSPRVE	7	939	945
bZIP	EVRFLRGKWQRRYRRIYDLIELCGSLHHIW QNLLQTEENLLDFVRFM	AEVRFLRGKWQRRFRRIFDLIELCGSLHHV WQNMLQTEENLLDFVRFM	29	263	291
RBP-Jk	TATLRTLTPVPNRVGADSIMLTATFGCQN AARTLNTFSATVWTPPHAGPREQERY	TATLRTLTPVPNRVGADSIMLTATFGCQN GALAINTFSATVWIPPPAGPREQERY	55	183	240
Nm23-H1	QLPQSTGRKPQCFWEMRAGREITQMQQEP SSHLQSATQP	LLTQSTGPAPRSFWEMRAGRDAPKIQQEPS SQQQPATQS	55	637	675

Supplementary Table S5. Recominations event identified by Gubbins. Bold and underlined recombination block numbers correspond to recombinations found only in EBV type 1, while other recombination numbers correspond to EBV type 2.

Recombination block	Begin	End	Isolates
**1**	2104	2154	KT823509 AP019150 AP019151 AP019112 AP019013 AP019061 AP019102 AP019040 AP019148 AP019114 AP019140 AP019067 AP019142 AP019111 AP019141 AP019080 AP019014 AP019104 AP019169 AP019166 AP019179 AP019162 AP019042 AP019051 AP019180 AP019036 AP019096 AP019060 AP019018 AP019030 AP019079 AP019124 AP019062 AP019070 KT823506 MH590485 MH590461 MH590526 MH590562 MH590460 KT823508 MH590404 MH590533 MH101965 MG021307 KT273949 MH590470 MH590383 AP019181 MG298846 MH590544 LR994495 MG298865 LR994477 MH590389 LR994480 LR994488 LR994487 HQ020558 LR994518 LR994490 LR994494 LR994478 LR994479 LR994501 LR994502 LR994486 LR994541 MH590523 MH590498 MH590406 MH590540 OM022194 MH590395 MH590578 MH590577 MH590519 MH590454 MH590520 MH590570 MH590423 MH590568 MH590456 MH590532 MH590541 OM022148 OM022197 OM022211 OM022225 MH590542 MH590474 OM022219 MH590538 MH590500 OM022126 MH590471 LR994503 KF992571 MH590447 OM022222 MH590553 MH590469 MH590516 MH590504 MH590557 LR994492 MH590414 OM022220 MH590534 LR994507 LR994489 LR994491 KF992570 OM022155 LR994519 OM022145 JQ009376 LR994533 OM022215 OM022214 LR994534 LR994513 OM022203 OM022191 MH590426 MH590403 MH590409 OM022187 MH590564 MH590433 MG298906 MH590524 MH590463 LR994537 MH590379 MH590531 MH590375 MH590386 MH590548 LR994523 MH590545 MH590566 OM022133 LR994476 OM022190 KX674064 KX125050 MH590407 MH590480 OM022127 MH590442 MH590515 KF992566 MH590522 MH590560 MH590535 MH590479 MH590559 KF373730 LR994520 OM022205 MG298896 LR994526 LR994510 MH590512 MH590416 OM022128 LR994532 MH590370 MH590565 MH590536 MH590552 MH590513 MH590434 MH590569 MH590527 MH590543 KF992565 MH590514 OM022212 KF992564 LR994516 LR994483 LR994529 OM022216 LR994536 OM022196 OM022154 LR994481 MH590521 MH590457 KF992567 MH101959 MH590575 KJ411974 KC617875 MH590408 LR994484 OM022198 OM022207 LR994521 OM022132 MH101962 OM022157 MH590528 OM022122 MH590432 KT273947 KT254013 MH590509 MH590525 KT273943 MH590529 MH590405 OM022226 MH590561 MH590427 KT273946 MH590555 MG298854 MH883785 LS992264 AP019049 AP019048 AP019065 AP019064 AP019087 AP019032 AP019053 AP019044 AP019172 AP019183 AP019182 AP019187 AP019118 MH590373 AP019028 AP019027 KC207813 AP019125 AP019135 AP019076 AP019083 AP019164 AP019029 AP019038 AP019108 AP019136 MH590445 23
**2**	2814	2886	MH590399 MG021316 MG298891 MG298886 MG298884 MG298876 MH144221 MG298880 MG298873 MG298890

			MG298885 MG298887 MG298882 MG298895 MG298874 MG298894 MG298875 MG298879 MH590550 MH590573 KX125051 KX674065 OM022125 OM022142 KT273945 OM022209 MH590384 MH590549 MG298858 MH590472 MG298823 MH590465 OM022221 MH590380 MH590391 MH590496 OM022185 OM022218 AP019025 LR994535 MH590475 LR994508 MH144213 MH590394 MH590459 MH590393 MH101968 MH101971 MG298824 MH101969 MH590491 MH590437 MH590495 MH101958 MH101970 MG298907 MG298916 MH590507 MH590418 MG298851 MH590402 MH590372 MG298912 MG298913 MG298908 MG298909 MG298910 MH590428 MH590511 OM022152 MH590420
3	2518	2653	MG298893 MT648642 MG298915 LR994522 MG298898 LR812996 LR813000 LR813051 LR812985 LR812990 LR812994 LR813079 LR813143 LR813063 LR812979 LR813042 LR813047 LR813019 LR813052 LR812997 LR813061 LR812981 LR812998 LR812983 LR813073 LR813001 LR813045 LR813040 LR813009 LR813034 LR813021 LR813069 LR813053 OR652420 MG298831 MT648657 OR652422 MG298923 NC009334 DQ279927 MG298827 MH590466 HZ437644 MG021312 MH590446 LC573552 MH590441 MH590467 LC573550 MH590448 MH590488 MB445486 MH590417 MH590492 OM022151 MH590473 MH590410 MH590422 MH590510 MH590421 MG298842 MH590576 MH590503 MH590556 MH590436 MG298911 MG298914 LR994527 MH144216 OM022129 MH590381 MH590508 OM022137 MH590452 OM022121 OM022206 MH590505
4	1777	2398	MG298893 MT648642 MG298915 LR994522 MG298898 LR812996 LR813000 LR813051 LR812985 LR812990 LR812994 LR813079 LR813143 LR813063 LR812979 LR813042 LR813047 LR813019 LR813052 LR812997 LR813061 LR812981 LR812998 LR812983 LR813073 LR813001 LR813045 LR813040 LR813009 LR813034 LR813021 LR813069 LR813053 OR652420 MG298831 MT648657 OR652422 MG298923 NC009334 DQ279927 MG298827 MH590466 HZ437644 MG021312 MH590446 LC573552 MH590441 MH590467 LC573550 MH590448 MH590488 MB445486 MH590417 MH590492 OM022151 MH590473 MH590410 MH590422 MH590510 MH590421 MG298842 MH590576 MH590503 MH590556 MH590436 MG298911 MG298914 LR994527 MH144216 OM022129 MH590381 MH590508 OM022137 MH590452 OM022121 OM022206 MH590505
5	1445	1570	MK540378 MK540402 MG298893 MT648642 MG298915 LR994522 MG298898 LR812996 LR813000 LR813051 LR812985 LR812990 LR812994 LR813079 LR813143 LR813063 LR812979 LR813042 LR813047 LR813019 LR813052 LR812997 LR813061 LR812981 LR812998 LR812983 LR813073 LR813001 LR813045 LR813040 LR813009 LR813034 LR813021 LR813069 LR813053 OR652420 MG298831 MT648657 OR652422 MG298923 NC009334 DQ279927 MG298827 MH590466 HZ437644 MG021312 MH590446 LC573552 MH590441 MH590467 LC573550 MH590448

			MH590488 MB445486 MH590417 MH590492 OM022151 MH590473 MH590410 MH590422 MH590510 MH590421 MG298842 MH590576 MH590503 MH590556 MH590436 MG298911 MG298914 LR994527 MH144216 OM022129 MH590381 MH590508 OM022137 MH590452 OM022121 OM022206 MH590505
6	71	246	MK540378 MK540402 MG298893 MT648642 MG298915 LR994522 MG298898 LR812996 LR813000 LR813051 LR812985 LR812990 LR812994 LR813079 LR813143 LR813063 LR812979 LR813042 LR813047 LR813019 LR813052 LR812997 LR813061 LR812981 LR812998 LR812983 LR813073 LR813001 LR813045 LR813040 LR813009 LR813034 LR813021 LR813069 LR813053 OR652420 MG298831 MT648657 OR652422 MG298923 NC009334 DQ279927 MG298827 MH590466 HZ437644 MG021312 MH590446 LC573552 MH590441 MH590467 LC573550 MH590448 MH590488 MB445486 MH590417 MH590492 OM022151 MH590473 MH590410 MH590422 MH590510 MH590421 MG298842 MH590576 MH590503 MH590556 MH590436 MG298911 MG298914 LR994527 MH144216 OM022129 MH590381 MH590508 OM022137 MH590452 OM022121 OM022206 MH590505
7	2654	2785	MK540378 MK540402 MG298893 MT648642 MG298915 LR994522 MG298898 LR812996 LR813000 LR813051 LR812985 LR812990 LR812994 LR813079 LR813143 LR813063 LR812979 LR813042 LR813047 LR813019 LR813052 LR812997 LR813061 LR812981 LR812998 LR812983 LR813073 LR813001 LR813045 LR813040 LR813009 LR813034 LR813021 LR813069 LR813053 OR652420 MG298831 MT648657 OR652422 MG298923 NC009334 DQ279927 MG298827 MH590466 HZ437644 MG021312 MH590446 LC573552 MH590441 MH590467 LC573550 MH590448 MH590488 MB445486 MH590417 MH590492 OM022151 MH590473 MH590410 MH590422 MH590510 MH590421 MG298842 MH590576 MH590503 MH590556 MH590436 MG298911 MG298914 LR994527 MH144216 OM022129 MH590381 MH590508 OM022137 MH590452 OM022121 OM022206 MH590505 MK540291 MK540382
8	1963	2094	MK540378 MK540402 MG298893 MT648642 MG298915 LR994522 MG298898 LR812996 LR813000 LR813051 LR812985 LR812990 LR812994 LR813079 LR813143 LR813063 LR812979 LR813042 LR813047 LR813019 LR813052 LR812997 LR813061 LR812981 LR812998 LR812983 LR813073 LR813001 LR813045 LR813040 LR813009 LR813034 LR813021 LR813069 LR813053 OR652420 MG298831 MT648657 OR652422 MG298923 NC009334 DQ279927 MG298827 MH590466 HZ437644 MG021312 MH590446 LC573552 MH590441 MH590467 LC573550 MH590448 MH590488 MB445486 MH590417 MH590492 OM022151 MH590473 MH590410 MH590422 MH590510 MH590421 MG298842 MH590576 MH590503 MH590556 MH590436 MG298911 MG298914 LR994527 MH144216 OM022129 MH590381 MH590508 OM022137 MH590452 OM022121 OM022206 MH590505 MK540291 MK540382

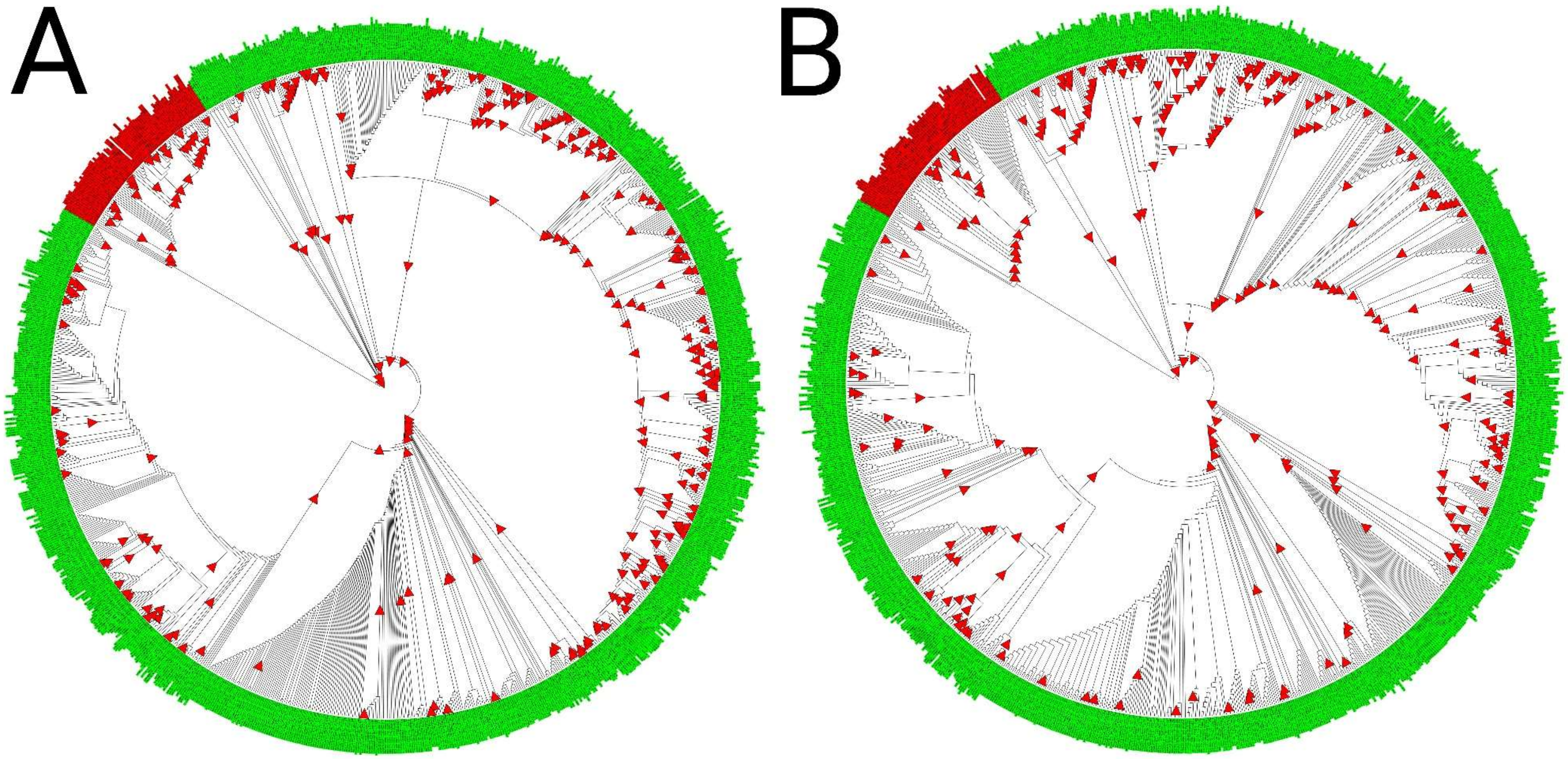

Supplementary Figure S1. Comparison of EBNA3C unfiltered vs. filtered recombinant regions. Maximum likelihood phylogenetic reconstruction of both trees includes BERF3 and BERF4 exons. Visualization using iTOL, the distance of branches was ignored, and the trees were rooted at the midpoint. (**A**) Phylogenetic tree without filtering the recombinant regions. (**B**) Phylogenetic tree with the recombinant regions detected filtered. Isolates with names in red correspond to EBV-2, and those in green correspond to EBV-1. Bootstrap values above 0.75 are shown with red triangles

Supplementary Table S6. List of Uniprot ID for each analyzed protein

Protein	N° Amino acids	Uniprot ID
EBNA3C type 1	992	P03204
EBNA3C type 2	1069	Q69140
